# Use of multi-flip angle measurements to account for transmit inhomogeneity and non-Gaussian diffusion in DW-SSFP

**DOI:** 10.1016/j.neuroimage.2020.117113

**Published:** 2020-10-15

**Authors:** Benjamin C. Tendler, Sean Foxley, Moises Hernandez-Fernandez, Michiel Cottaar, Connor Scott, Olaf Ansorge, Karla L. Miller, Saad Jbabdi

**Affiliations:** a Wellcome Centre for Integrative Neuroimaging, FMRIB, Nuffield Department of Clinical Neurosciences, University of Oxford, Oxford, UK; b Department of Radiology, University of Chicago, Chicago, IL, USA; c Centre for Biomedical Image Computing and Analytics, University of Pennsylvania, PA, USA; d Nuffield Department of Clinical Neurosciences, University of Oxford, Oxford, UK

**Keywords:** Post-mortem human brain, Diffusion-weighted steady-state free precession, 7T, Diffusion tensor imaging, B_1_ inhomogeneity, b-value

## Abstract

Diffusion-weighted steady-state free precession (DW-SSFP) is an SNR-efficient diffusion imaging method. The improved SNR and resolution available at ultra-high field has motivated its use at 7T. However, these data tend to have severe B_1_ inhomogeneity, leading not only to spatially varying SNR, but also to spatially varying diffusivity estimates, confounding comparisons both between and within datasets. This study proposes the acquisition of DW-SSFP data at two-flip angles in combination with explicit modelling of non-Gaussian diffusion to address B_1_ inhomogeneity at 7T. Data were acquired from five fixed whole human post-mortem brains with a pair of flip angles that jointly optimize the diffusion contrast-to-noise (CNR) across the brain. We compared one- and two-flip angle DW-SSFP data using a tensor model that incorporates the full DW-SSFP Buxton signal, in addition to tractography performed over the cingulum bundle and pre-frontal cortex using a ball & sticks model. The two-flip angle DW-SSFP data produced angular uncertainty and tractography estimates close to the CNR optimal regions in the single-flip angle datasets. The two-flip angle tensor estimates were subsequently fitted using a modified DW-SSFP signal model that incorporates a gamma distribution of diffusivities. This allowed us to generate tensor maps at a single effective b-value yielding more consistent SNR across tissue, in addition to eliminating the B_1_ dependence on diffusion coefficients and orientation maps. Our proposed approach will allow the use of DW-SSFP at 7T to derive diffusivity estimates that have greater interpretability, both within a single dataset and between experiments.

## Highlights

• B_1_ inhomogeneity at 7T leads to spatially varying SNR & ADC estimates in DW-SSFP.
• 2-flip angle DW-SSFP data can address B_1_ effects in a cohort of post-mortem brains.
• Our approach reduces degradations in PDD estimates & improves whole brain coverage.
• Our approach improves tractography performance across areas of high and low B_1_.
• Our approach provides a means to define ADCs at a single effective b-value.

## 1 Introduction

Diffusion imaging of post-mortem human brains has important applications for both validating diffusion contrast mechanisms through comparison with microscopy and achieving very high-resolution data with long scan times. However, post-mortem diffusion imaging presents significant challenges due to changes in tissue properties related to death and fixation. Unfavorable reductions in T_1_, T_2_ and diffusion coefficient have been observed in fixed tissue using a variety of fixation methods (Blamire et al., 1999; D’Arceuil and de Crespigny, 2007; Dawe et al., 2009; Shepherd et al., 2009; Yong-Hing et al., 2005).

One method to overcome these changes is to utilize an imaging strategy that allows for fast acquisition of the MR signal to overcome the losses associated with the shortened T_2_ values. We have previously proposed the use of diffusion-weighted steady-state free precession (DW-SSFP) for post-mortem imaging due to its ability to achieve robust signal and strong diffusion contrast in short-T_2_ species (McNab et al., 2009). The high signal-to-noise (SNR) efficiency of DW-SSFP compared to diffusion-weighted spin echo (DW-SE) acquisitions enables improvements in the quality of both diffusion tractography and estimates of multiple fiber populations at 3T (Miller et al., 2012), motivating its use in post-mortem samples (Berns et al., 2015; Cardenas et al., 2017; Pallebage-Gamarallage et al., 2018; Vasung et al., 2019; Wilkinson et al., 2016).

Ultra-high field scanners have potential to enable further gains in spatial resolution, with DW-SSFP providing a valuable tool for addressing the even shorter T_2_ values at 7T and above (Foxley et al., 2014a). However, DW-SSFP data acquired at 7T are compromised by B_1_ inhomogeneity. This presents us with a challenge: unlike other diffusion imaging sequences, both the signal and diffusion attenuation in DW-SSFP are sensitive to flip angle (Buxton, 1993). The DW-SSFP signal is sensitive to the effects of restriction (McNab and Miller, 2008), and in systems with non-Gaussian diffusion (due to restrictions in tissue), this leads to diffusivity estimates that can depend on the applied flip angle.

Given a B_1_ field map, we propose an approach to account for these issues by acquiring a pair of DW-SSFP datasets at two different flip angles. This dual-flip angle approach has two advantages: Firstly, our flip angles can be chosen such that different regions of tissue have high SNR in the individual datasets (Foxley et al., 2014b). We can subsequently combine the datasets in a manner to yield high SNR diffusivity estimates over the entire brain. Secondly, we can modify the DW-SSFP signal equation to account for how the measured apparent diffusion coefficient (ADC) varies with flip angle under a simple model of non-Gaussian diffusion. From this, we can explicitly model the relationship between the effective b-value and flip angle using a previously described framework (Tendler et al., 2020). Here we describe a method to subsequently derive diffusivity estimates over the entire brain sample interpolated to a single effective b-value, removing the influence of B_1_.

## 2 Theory

### 2.1 Dual-flip angle acquisition to optimize diffusion contrast

**Fig. 1:**
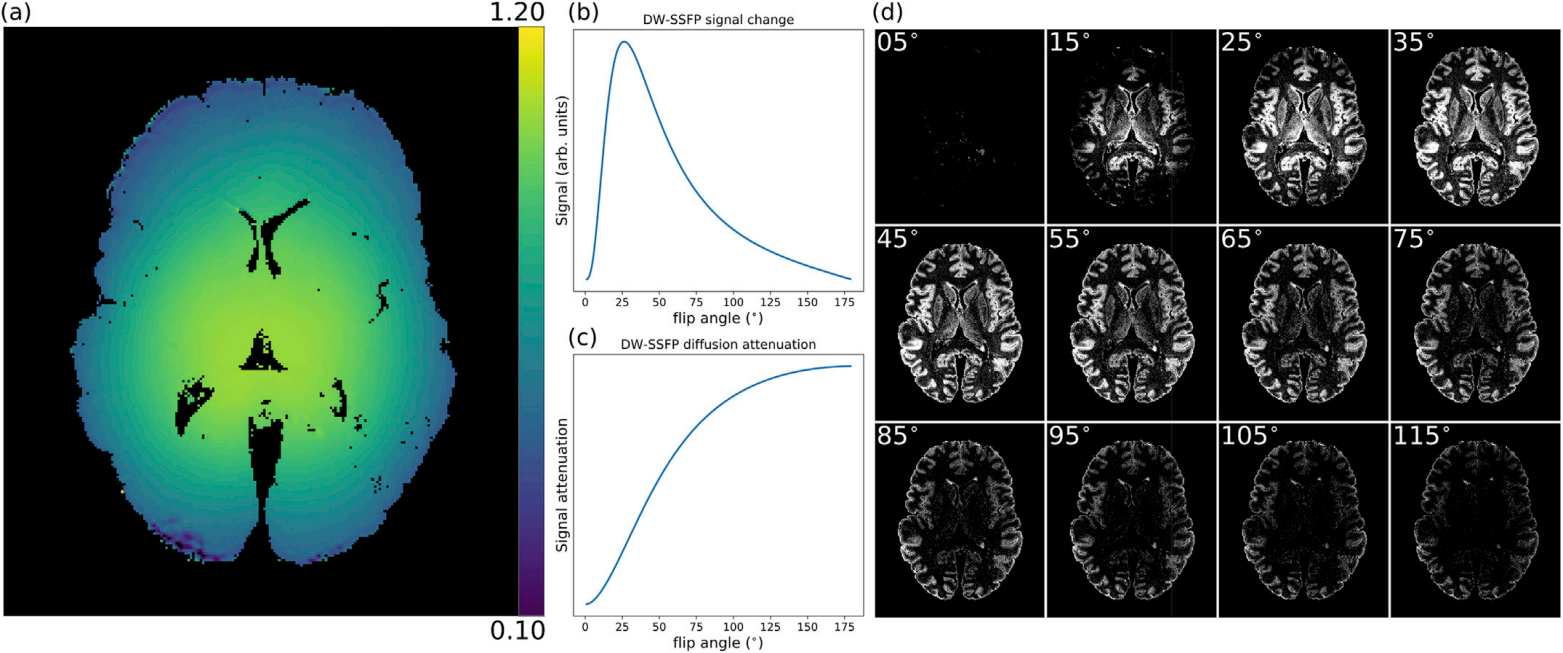
**B**_**1**_**inhomogeneities at 7T.** (a) A single slice of a B_1_ map estimated using the method described in (Yarnykh, 2007) obtained over a whole post-mortem human brain sample at 7T. B_1_ decreases smoothly as we approach the edge of the brain. The signal (b) and diffusion attenuation (c) have a strong flip angle dependence in DW-SSFP. (d) Example DW-SSFP images acquired with multiple nominal flip-angles at 7T reveal how a change in flip-angle yields changes in signal, with the impact of the nominal flip angle and B_1_ clearly visible between and within DW-SSFP datasets, consistent with the signal simulation in (b). As the nominal flip angle increases, a bright concentric ring in the DW-SSFP data moves radially towards the edge of the brain. (b) and (d) depict the DW-SSFP signal change (CNR).

At ultra-high field, B_1_ inhomogeneity (Fig. 1a) leads to a spatially varying flip angle across the brain, where in DW-SSFP both the measured signal (Fig. 1b) and diffusion attenuation (Fig. 1c) are sensitive to the applied flip angle. Through appropriate setting of the RF transmit gain, one can control where in the brain a desired flip angle is achieved. In practice, the simplest way to achieve this is to adjust the nominal flip angle (i.e. the flip angle specified on the scanner console). This effect is demonstrated in Fig. 1d, which displays a single slice through a DW-SSFP dataset where the nominal flip angle is changed by 10° increments from 5° to 115°. By changing the nominal flip angle, a bright concentric ring is seen to move radially from the centre of the brain towards the edge.

**Fig. 2:**
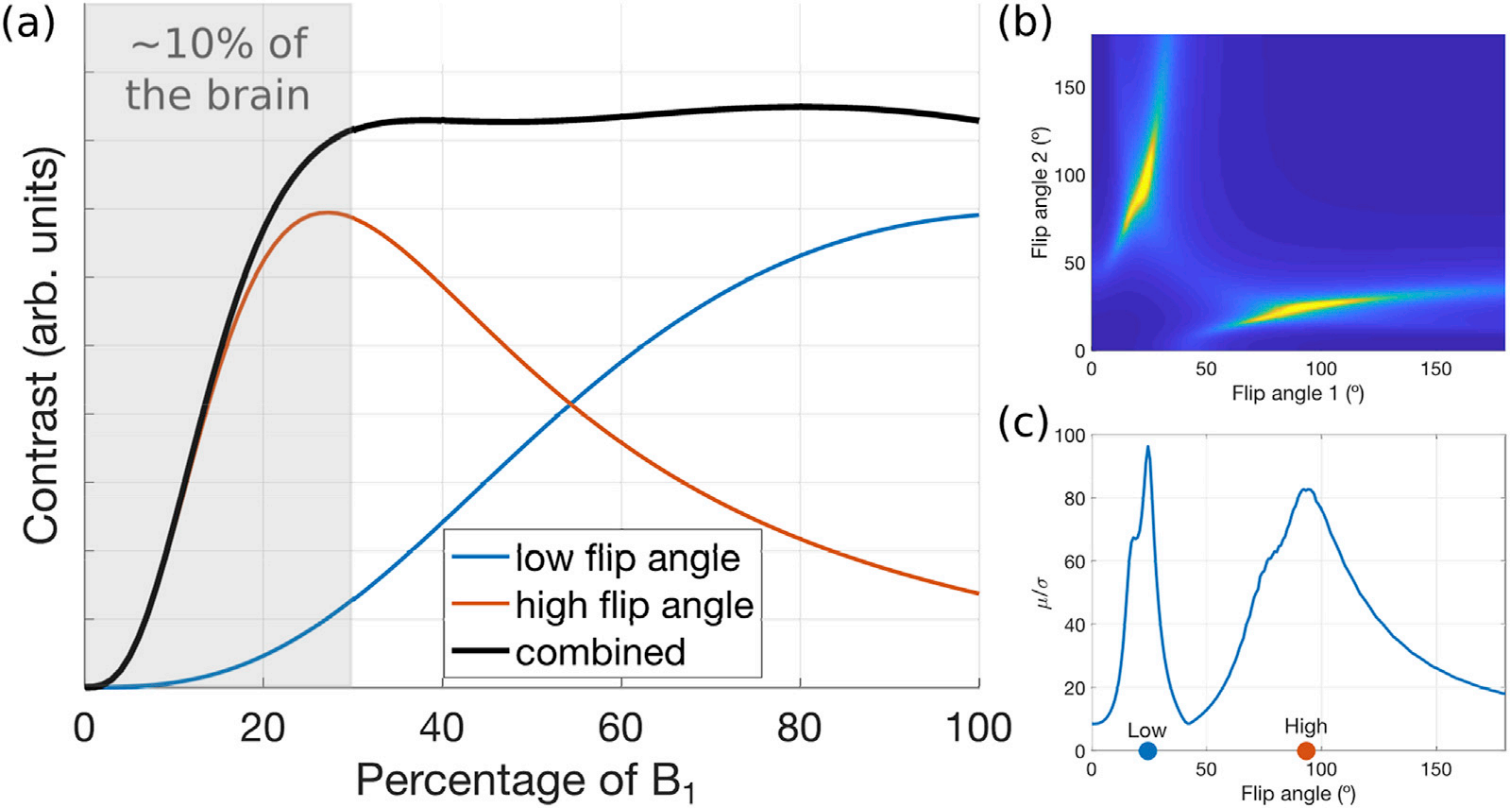
**Optimization used for the two-flip angle DW-SSFP acquisition.** The red and blue curves in (a) show the variation in diffusion contrast (difference of diffusion weighted and non-diffusion-weighted signal) across the brain as a function of B_1_. We combined the contrast curves across pairs of flip angles to maximize the quantity μ/σ (b), where μ is the mean contrast across the B_1_ range and σ is the standard deviation. This metric aims for maximum contrast with minimum variation across the brain (black curve on the left). Here, we only considered a range of 30%–100% of maximum B_1_ for our calculations of μ and σ which corresponds to ~90% of the brain, so as not to have the optimization dominated by a minority of brain voxels where contrast changes rapidly with flip angle. Our simulations estimated a CNR-optimal flip angle pair of 24° and 94° (c). Simulation parameters were approximately matched to our protocol at 7T: $T_{1}=500\;\text{ms}$, $T_{2}=30\;\text{ms}$, $\text{ADC}=1\cdot 10^{-4}\;{\text{mm}}^{2}/\text{s}$, TR=30ms, diffusion gradient amplitude=52mT/m, diffusion gradient duration=14ms.

An arbitrarily optimized flip angle for the DW-SSFP signal can therefore be predictably positioned with knowledge of B_1_. We propose that the signal dependency on B_1_ can be mitigated by acquiring data with an optimized pair of flip angles. Fig. 2 outlines our proposed optimization procedure, which aims to produce high contrast across the entire brain. The goal is to identify an optimal pair of nominal flip angles based on the predicted diffusion contrast (here defined as the difference between the non-diffusion and diffusion weighted signals). An ideal flip angle pair would achieve both high and homogeneous contrast over a large range of B_1_ (Fig. 2). To achieve this, DW-SSFP contrast curves were generated for every pair of flip angles (Fig. 2a) and their mean (μ) and standard deviation (σ) over a range of B_1_ values were determined. To identify a flip angle pair that represented a balance of high contrast and homogeneity across a range of B_1_, we calculated the variance-normalized mean (μ/σ) of all flip angle pairs (Fig. 2b), and chose the peak value as our optimal pair of flip angles (Fig. 2c). We considered a range of 30–100% of the maximum B_1_ (Fig. 2a) to ensure that the optimization is not dominated by a minority of voxels with very low B_1_.

### 2.2 A DW-SSFP effective b-value

**Fig. 3:**
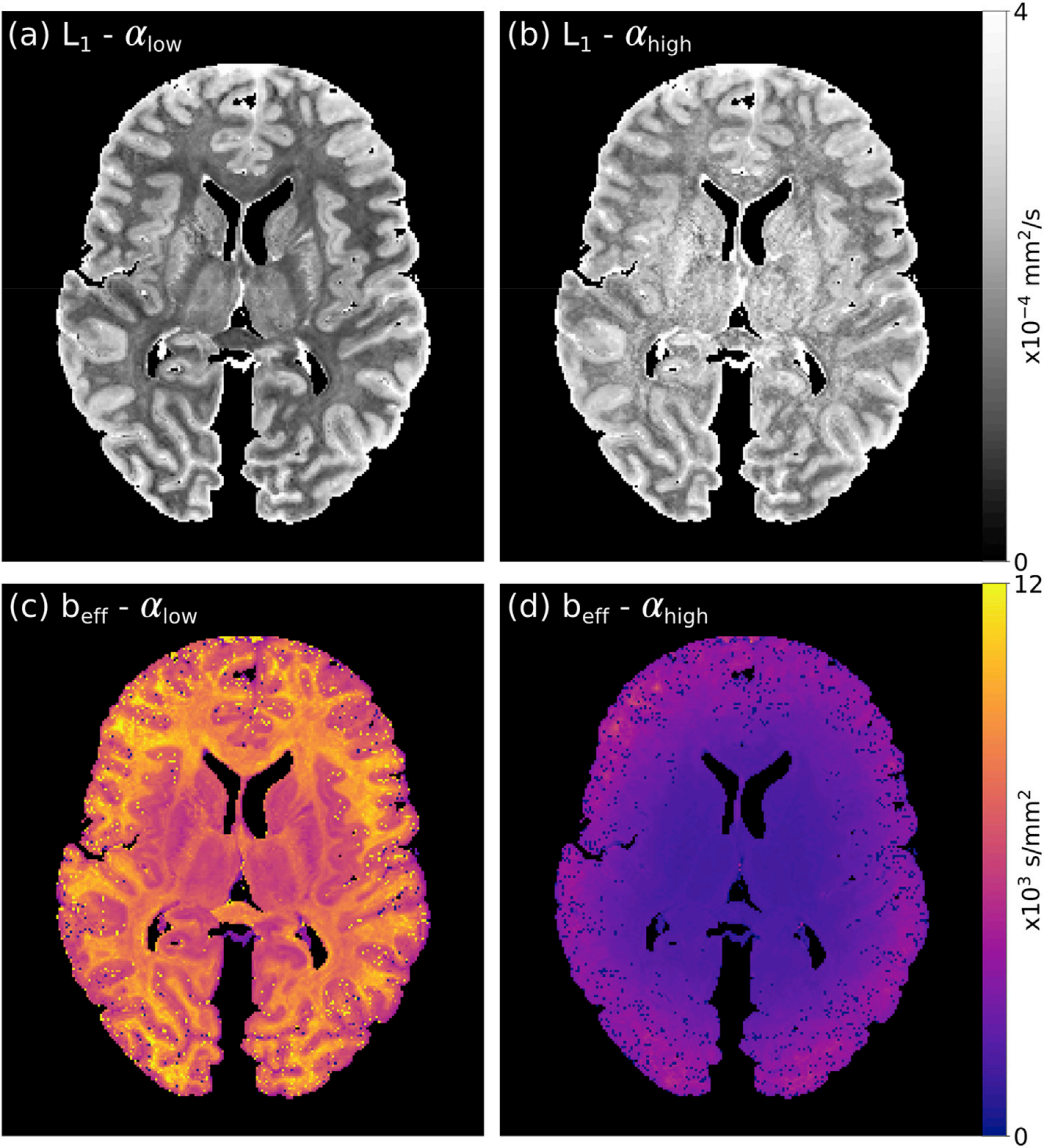
**Flip angle dependency of diffusivity estimates in DW-SSFP.** (a) and (b) display estimated principal eigenvalue ($L_{1}$) maps from DW-SSFP data acquired at a low ($\alpha_{\text{low}}$) and high ($\alpha_{\text{high}}$) nominal flip angle in a whole post-mortem human brain sample. The diffusion coefficients estimated at $\alpha_{\text{high}}$ are higher than those estimated at $\alpha_{\text{low}}$, highlighting that in DW-SSFP, changing the flip angle is analogous to acquiring data at a different effective b-value (${\text{b}}_{\text{eff}}$). At 7T, B_1_ inhomogeneity leads to a spatially-varying flip angle across the brain (Fig. 1a). This further translates into a spatially varying effective b-value (${\text{b}}_{\text{eff}}$) across tissue. The ${\text{b}}_{\text{eff}}$ maps displayed in (c) and (d) reveal that we estimate a higher ${\text{b}}_{\text{eff}}$ at a reduced flip angle, with ${\text{b}}_{\text{eff}}$ increasing as we approach the brain boundary (in regions of low B_1_). In DW-SSFP, the T_1_ and T_2_ of tissue additionally influence ${\text{b}}_{\text{eff}}$, leading to grey/white matter contrast in the ${\text{b}}_{\text{eff}}$ maps. $L_{1}$ maps derived using a tensor model from ‘Brain 1’ as described in the Methods section.

In systems with non-Gaussian diffusion (due to restrictions in tissue), the ADC is dependent on the applied flip angle in DW-SSFP (Fig. 3a and b). The dependence of ADC on the flip-angle is problematic, as variations in the applied flip angle across a single brain (due to B_1_ inhomogeneity) amounts to having different b-values in different parts of the image (Fig. 3c and d). This leads to spatially-dependent ADC estimates within a single DW-SSFP dataset (Fig. 3 and (Tendler et al., 2020)), analogous to acquiring a dataset with different b-values across the brain with a standard DW-SE experiment.

**Fig. 4:**
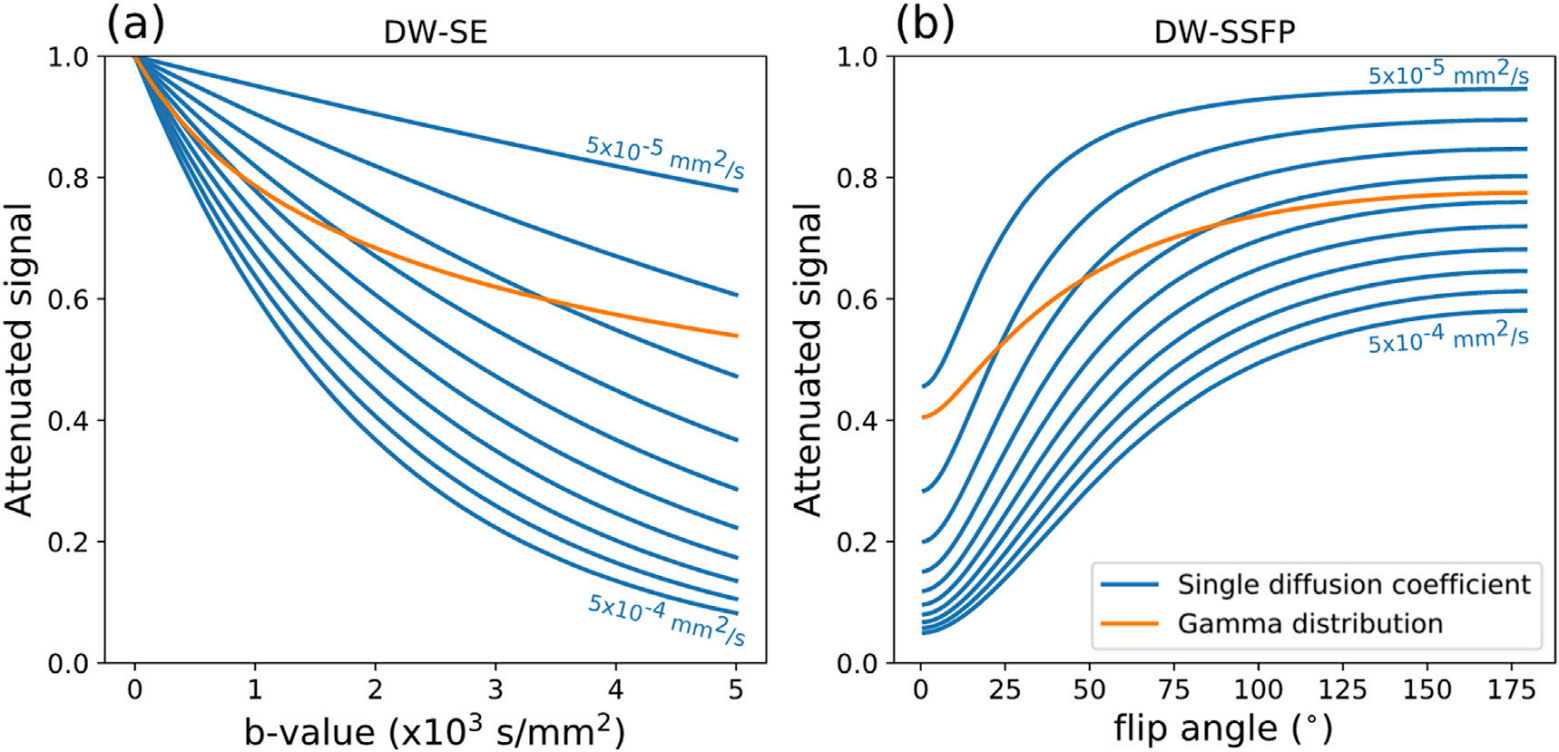
**Simulating non-Gaussian diffusion effects on ADC.** Comparison of the diffusion attenuation of a multi-b-value DW-SE (a) and multi-flip angle DW-SSFP (b) experiment in a system defined by a single diffusion coefficient (Gaussian diffusion – blue lines) or non-Gaussian diffusion (gamma distribution of diffusivities - orange line). Here each blue line represents a different diffusion coefficient, ranging from $D=5\cdot 10^{-5}$ (top) to $5\cdot 10^{-4}$ mm^2^/s (bottom). Under Gaussian diffusion (blue lines), the diffusion attenuation curves do not overlap for different diffusion coefficients. However, for non-Gaussian diffusion (orange lines), the diffusion attenuation curves cross through the blue lines, indicating a change in the measured ADC.

This effect is illustrated in Fig. 4, which simulates multi-b-value DW-SE and multi-flip angle DW-SSFP diffusion attenuation for systems defined by a single diffusion coefficient (Gaussian) vs a gamma distribution of diffusivities (non-Gaussian). The attenuation curve of the gamma model (orange line) crosses the constant-ADC curves of the Gaussian model (blue lines) when changing both b-value (in DW-SE) and flip angle (in DW-SSFP), implying a change in the measured ADC.

However, the ADC is not only a function of flip angle in DW-SSFP, but is also modulated by relaxation (Fig. 3). One way to view this is to think of the DW-SSFP signal as a linear mixture of coherence pathways with different b-values. The relative weights of the pathways are determined by the flip angle, but also by the relaxation times T_1_ and T_2_, leading to a tissue-dependent effective b-value affecting the ADC estimates. It is worth emphasising that for non-Gaussian diffusion systems, one cannot solve this problem by measuring T_1_ and T_2_ and incorporating these estimates into the DW-SSFP signal (modelling with a single ADC), since the b-value will still be influenced by relaxation (Fig. 3c).

Recently, we have proposed an approach (detailed in (Tendler et al., 2020)) that defines ADC estimates from DW-SSFP as a function of an effective b-value, ${\text{b}}_{\text{eff}}$. This definition is able to account for the effects of flip angle and relaxation on DW-SSFP ADC estimates, in contrast to previous work (Miller et al., 2012), which defined ${\text{b}}_{\text{eff}}$ in terms of the DW-SSFP diffusion attenuation. We achieve this by explicitly incorporating models of non-Gaussian diffusion into the DW-SSFP signal equations, and making comparisons with the DW-SE ADC predictions under the same model of non-Gaussianity. Below we describe how we can use this approach to correct for the influence of variable effective b-values across tissue (Fig. 3c and d), to generate ADC estimates at the same effective b-value across the entire brain from DW-SSFP data acquired at two flip angles.

### 2.3 Generating DW-SSFP estimates at a single effective b-value across the entire brain

DW-SSFP data acquired at two flip angles will lead to distinctive diffusivity estimates at each flip angle when considering a non-Gaussian system (Fig. 3a and b). To account for this, we can fit an extension to the DW-SSFP signal model that incorporates non-Gaussianity, to estimate the non-Gaussian system that is able to explain the variation in diffusivity with flip angle (Tendler et al., 2020). Here we use a non-Gaussian system described by a gamma distribution of diffusivities incorporated into the Buxton signal model of DW-SSFP (Buxton, 1993):

[1] $$\;S_{SSFP,\text{Γ}}\left(\alpha ,T_{1},T_{2},\text{TR},q,D_{m},D_{s}\right)=\int_{0}^{\infty }S_{SSFP}\left(\alpha ,T_{1},T_{2},\text{TR},q,D\right)\rho \left(D;D_{m},D_{s}\right)\;dD,$$

where the $S_{SSFP}$ is the Buxton DW-SSFP signal model (defined in Appendix Eq. [A1]) and $\rho \left(D;D_{m},D_{s}\right)$ is the gamma probability density function (PDF) with mean and standard deviation $D_{m}$ and $D_{s}$. This integral can be evaluated numerically.

We can subsequently use the voxelwise estimated gamma parameters to simulate the DW-SE signal under the same gamma PDF voxelwise (integral here is analytic) (Jbabdi et al., 2012; Oshio et al., 2014):

[2] $$\begin{array}{cc} S_{\text{SE},\text{Γ}}\left(b,D_{m},D_{s}\right) & =S_{0}\int_{0}^{\infty }e^{-bD}\rho \left(D;D_{m},D_{s}\right)\;dD\\ & =S_{0}{\left(\frac{D_{m}}{D_{m}+bD_{s}^{2}}\right)}^{\frac{D_{m}^{2}}{D_{s}^{2}}}. \end{array}$$

Given a target b-value (${\text{b}}_{\text{eff}}$), we can then define the ADC within every voxel at the same ${\text{b}}_{\text{eff}}$ by resolving:

[3] $$\begin{array}{cc} D\left({\text{b}}_{\text{eff}},D_{m},D_{s}\right) & =-\frac{1}{{\text{b}}_{\text{eff}}}\ln\left(\frac{S_{\text{SE},\text{Γ}}\left({\text{b}}_{\text{eff}},D_{m},D_{s}\right)}{S_{0}}\right)\\ & =-\frac{1}{{\text{b}}_{\text{eff}}}\cdot \frac{D_{m}^{2}}{D_{s}^{2}}\cdot \ln\left(\frac{D_{m}}{\left(D_{m}+{\text{b}}_{\text{eff}}D_{s}^{2}\right)}\right). \end{array}$$

**Fig. 5:**
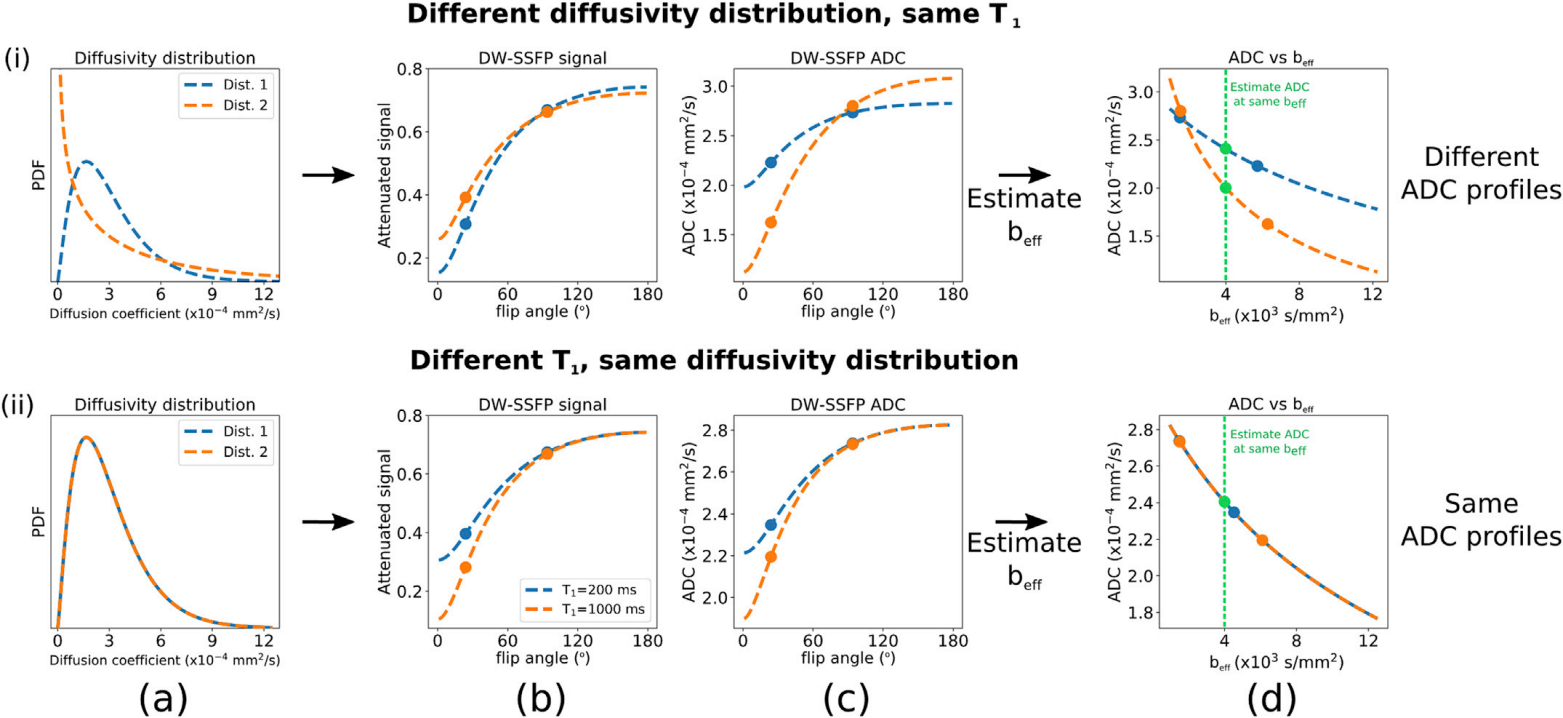
**Outline of the processing pipeline to generate ADC estimates at a single**$b_{eff}$**.** Here we consider two simulations of two voxels. In (i), the two voxels have different gamma PDFs describing non-Gaussianity (a), but identical relaxation properties. In (ii), the two voxels have identical gamma PDFs (a), but different T_1_ relaxation times. The simulated DW-SSFP signal varies as a function of flip angle (b), which gives rise to flip angle dependent ADCs (c) (if you assume Gaussianity and calculate a single ADC). From DW-SSFP data acquired at two flip angles (dots), we can estimate the diffusivity distributions (a) that are able to explain this variation with flip angle (b and c dotted lines) using Eq. [1]. If one is interested in characterising the non-Gaussianity, this step would provide us with the relevant characterization, as described in (Tendler et al., 2020). Here, however, we aim to instead translate the system into the equivalent measurement that would be made with DW-SE at a single well-defined effective b-value under the same model of non-Gaussian diffusion. Using Eq. [3], we can subsequently simulate the ADC as a function of effective b-value (d). From this, we can estimate the ADCs at a single ${\text{b}}_{\text{eff}}$ for all voxels (green line - ${\text{b}}_{\text{eff}}=4000\;{\text{mm}}^{2}/\text{s}$). In (i), the two gamma PDFs lead to distinctive ADC flip angle curves (c). However in (ii), the different T_1_ relaxation times additionally leads to distinctive ADC flip angle curves (c), where the parameters chosen lead to ADC estimates that appear identical at high flip angles, but diverge at lower flip angles, despite these measurements reflecting the same system of diffusivities (ii - a). Estimating the gamma PDF and plotting in terms of ${\text{b}}_{\text{eff}}$ leads to distinctive ADC ${\text{b}}_{\text{eff}}$ curves for (i), but identical curves for (ii).

Fig. 5 shows how these simple steps can recover the correct ADCs for measurements with different diffusivity distributions and relaxation times, thus removing both the potential tissue-type dependence as well as the flip-angle dependence.

Finally, we note that the choice of a gamma distribution to capture non-Gaussianity was motivated by its parsimony compared to e.g. a multi-exponential model, and thus we can fit this model with a minimum of two flip angles (a viable alternative could be to use kurtosis).

### 2.4 Extension to a tensor model

In this manuscript, we use a diffusion tensor model to evaluate our two flip angle approach and define our diffusivity estimates at a single effective b-value. To extend this approach to the diffusion tensor model (implementation for the Buxton model provided in Appendix Eq. [A2]), a slightly different procedure was adopted. While one could fit the tensor model to DW-SSFP data acquired at two flip angles directly (provided T_1_, T_2_, and B_1_ maps), the presence of non-Gaussianity renders the model inadequate, as the tensor eigenvalues are dependent on flip-angle and relaxation (leading to different eigenvalues for the DW-SSFP data acquired at each flip angle). The model needs to be augmented. However, there isn’t a simple but principled method to interpolate between the tensors fit at two flip angles, as this would have to assume some form of covariance. The principled approach would be to use a kurtosis tensor which explicitly fits this covariance (Jensen et al., 2005; Lu et al., 2006), but this introduces many more free parameters and would require measurements at many flip-angles as well as many directions.

Our approach to this is to estimate non-Gaussianity only along the tensor eigenvectors (again using the gamma model, but other models can be used). However, since the gamma model is only defined along the eigenvectors, we first perform a tensor-like fit to the data from the two flip angles, to estimate a single set of shared eigenvectors (${\vec{V}}_{1,2,3}$), but distinct eigenvalues ($L_{1,2,3}$ – a set estimated at each flip angle). We then estimate the parameters of the gamma distribution that best account for the change in the pairs of eigenvalues (i.e. fit a gamma to the two fitted $L_{1}$ values, a second gamma to the two fitted $L_{2}$ values, etc). Since this last fitting step only effectively uses data acquired at two flip angles (two eigenvalues), we regularise it by adding a prior on the mean of the gamma distribution as follows (e.g. assuming fitting along eigenvector ${\vec{V}}_{i}$):

[4] $$\;\underset{D_{m_{i}},D_{s_{i}}}{\text{ ​min}}{\left\|L_{i_{\text{sim}:\alpha_{\text{low}}}\;}\left(D_{m_{i}},D_{s_{i}}\right)-L_{i_{\text{exp}:\alpha_{\text{low}}}}\right\|}_{2}^{2}+{\left\|L_{i_{\text{sim}:\alpha_{\text{high}}}\;}\left(D_{m_{i}},D_{s_{i}}\right)-L_{i_{\text{exp}:\alpha_{\text{high}}}}\right\|}_{2}^{2}\;+\lambda {\left\|D_{m_{i}}-L_{{\text{i}}_{\text{exp}:\alpha_{\text{high}}}}\right\|}_{2}^{2}$$

where $\alpha_{\text{low}}$ and $\alpha_{\text{high}}$ are the voxelwise DW-SSFP flip angles, $L_{i_{\text{exp}:\alpha_{\text{low}}}}$/$L_{i_{\text{exp}:\alpha_{\text{high}}}}$ are the voxelwise experimental $L_{i}$ estimates at each flip angle, $L_{i_{\text{sim}:\alpha_{\text{low}}}\;}$/$L_{i_{\text{sim}:\alpha_{\text{high}}}\;}$ are the simulated $L_{i}$ estimates for a given $D_{m_{i}}$ and $D_{s_{i}}$ at each flip angle (estimated from Eq. [1] and Eq. [A1]) and λ is the regularization parameter. Finally, we then estimate an equivalent tensor at a chosen effective b-value as above using the spin-echo model (Eq. [3]), given estimated gamma distribution parameters along each eigenvector.

### 2.5 Extension to a ball & sticks model

To assess the performance of our dual flip acquisition vs a single flip angle, tractography was performed using a ball & sticks model (implementation for the Buxton model provided in Appendix Eq. [A3] and Eq. [A4]). To assess tractography performance, we simply fit two diffusivities for the data acquired at each flip angle, as we are only interested in the orientation of the sticks to perform tractography.

## 3 Methods

### 3.1 Sample preparation

Data were acquired in post-mortem human brains (n ​= ​5), comprised of two control brains and three brains from patients diagnosed with amyotrophic lateral sclerosis (ALS). Brains were extracted from the skull within 72 ​h after death. All brains were fixed for at least 45 days prior to scanning, with four brains fixed in 10% PBS buffered formalin and one brain fixed in 10% formalin (Brain 3). Prior to scanning, brains were removed from formalin and submerged in a perfluorocarbon liquid (Fluorinert FC-3283, 3M). The study was conducted under the Oxford Brain Bank’s generic Research Ethics Committee approval (15/SC/0639).

### 3.2 MRI data acquisition protocol

Data were obtained over the entire brain of each post-mortem sample on a human 7T Siemens whole body scanner (32ch-receive/1ch-transmit head coil). For each brain, DW-SSFP datasets were acquired at two nominal flip angles (24° and 94°), chosen based on the optimization described above. At each flip angle, 120 diffusion directions (q ​= ​300 ​cm^−1^) and six non-diffusion weighted datasets were acquired (resolution ​= ​0.85·0.85·0.85 ​mm^3^), with the same set of directions for both flip angles. Here we note the distinction between the nominal flip angles (24° and 94°) and the applied voxelwise flip angles ($\alpha_{\text{low}}$ and $\alpha_{\text{high}}$), which incorporate the effects of B_1_. These datasets will be subsequently referred to as $\alpha_{\text{low}}$ and $\alpha_{\text{high}}$ to highlight the change in flip angle across the brain.

To prevent banding artefacts in the non-diffusion weighted datasets, a slight diffusion gradient was applied along (x,y,z) = (0.557,0.577,0.577) to serve as a spoiler (q ​= ​20 ​cm^−1^) (Zur et al., 1988). In the case of non-diffusion weighed data, the Buxton model describes a reverse fast imaging with steady-state free procession (PSIF) sequence, which accounts for these spoiler gradients. These spoiler gradients lead to a negligible amount of diffusion weighting and are rarely taken into account in more conventional diffusion imaging (e.g. DW-SE). Due to this, these datasets were treated as non-diffusion weighted.

To aid in DW-SSFP quantification, we also acquired: B_1_ maps with an actual flip angle (AFI) acquisition (Yarnykh, 2007); T_1_ maps from a turbo inversion-recovery (TIR) sequence; and T_2_ maps from a turbo spin-echo (TSE) sequence. Full details of the acquisition protocol are provided in Table 1.

**Table 1:** **MRI imaging parameters.** The imaging parameters of the DW-SSFP dependency acquisitions (AFI, TIR and TSE) are representative of the parameters used, small modifications were made to these acquisitions as protocols evolved.

DW-SSFP		Turbo inversion-recovery (TIR)	
q-value (cm^−1^)	300	Resolution (mm^3^)	0.9·0.9·0.9
Diffusion Gradient Duration (ms)	13.56	Number of inversions	6
Diffusion Gradient Strength (mTm^−1^)	52	TE (ms)	14
Flip angles (^o^)	24 and 94	TR (ms)	1000
No. directions (per flip angle)	120	TIs (ms)	30, 60, 120, 240, 480 & 935
No. non-DW (per flip angle)	6 (q ​= ​20 ​cm^−1^)	Flip angle (^o^)	180
Resolution (mm^3^)	0.85·0.85·0.85	GRAPPA acc. factor	3
TE (ms)	21	Bandwidth (Hz per pixel)	130
TR (ms)	28	Acquisition time (per TI)	40:49
EPI factor	3	Number of averages	1
Bandwidth (Hz per pixel)	393		
Acquisition time (per direction/non-DW)	5:47	Turbo spin-echo (TSE) – T_2_	
Acquisition time (per flip angle)	12:08:42	Resolution (mm^3^)	0.9·0.9·0.9
No. of averages	1	Number of echoes	6
		TEs (ms)	13, 25, 38, 50, 63 & 76
Actual flip-angle imaging (AFI) – B_1_		TR (ms)	1000
Resolution (mm^3^)	3·3·3	Flip angle (^o^)	180
TE (ms)	1.5	GRAPPA acc. factor	2
TR_1_/TR_2_ (ms)	4.4/11	Bandwidth (Hz per pixel)	166
Flip angle (^o^)	60	Acquisition time (per TE)	36:01
Bandwidth (Hz per pixel)	630	Number of averages	1
Acquisition time	0:41		
Number of averages	1		

### 3.3 Data Processing

All coregistration between and within imaging modalities were performed with a 6 degrees-of-freedom (translations and rotations) co-registration via FLIRT (Jenkinson et al., 2002; Jenkinson and Smith, 2001). A Gibbs ringing correction was performed on the DW-SSFP, TIR and TSE datasets (Kellner et al., 2016). T_1_ and T_2_ maps were generated from the TIR and TSE data via a voxelwise fit assuming mono-exponential signal evolution. B_1_ maps were generated from the AFI datasets via the processing outlined in the original publication (Yarnykh, 2007) All data were processed and analyzed using the FMRIB software library (FSL) (Jenkinson et al., 2012) and Python (Millman and Aivazis, 2011). A tensor model (details in Appendix Eq. [A2]) that incorporates the full DW-SSFP Buxton signal model (Buxton, 1993) was fitted to the DW-SSFP data using cuDIMOT (Hernandez-Fernandez et al., 2019). When fitting the tensor, the order of the eigenvalues ($L_{1,2,3}$) was preserved with the constraint $L_{1}>L_{2}>L_{3}>0$. Voxelwise estimates of T_1_, T_2_ and B_1_ were incorporated into the model as fixed parameters. To eliminate bias in low signal regions due to the noise-floor (Jones and Basser, 2004), the mean DW-SSFP background signal was estimated and incorporated into the fitting (Gudbjartsson and Patz, 1995) using:

[5] $$S={\left(S_{SSFP}^{2}+S_{nf}^{2}\right)}^{0.5},$$

where $S_{SSFP}$ is the DW-SSFP Buxton signal model and $S_{nf}$ is the noise-floor estimate, set as a fixed parameter.

This work incorporates two versions of the diffusion tensor model, one which fits to DW-SSFP data acquired at one-flip angle and one that fits to data acquired at two-flip angles simultaneously. For the diffusion tensor, the latter analysis outputs a unique set of eigenvalues ($L_{1,2,3}$) for the DW-SSFP data acquired at each flip angle, but is constrained to a shared set of eigenvectors (${\vec{V}}_{1,2,3}$).

All comparative analyses were done solely over white matter, with white matter masks generated using FAST (Zhang et al., 2001) (masks displayed in Supplementary Material Fig. S1). When fitting to the DW-SSFP data acquired at two flip angles, an additional constraint was applied to prevent spurious diffusivity estimates in regions of very low signal (Supplementary Material: *Constraint for the dual-flip approach due to regions of low signal*).

### 3.4 Comparison of PDD estimates acquired with one- and two-flip angle acquisitions

To compare the resulting diffusion tensor eigenvectors between the one- and two-flip angle acquisitions, a time-matched comparison was performed. A subset of the data (60 directions at each flip angle) were selected and fitted with the two-flip angle DW-SSFP tensor model described above. These model fits were compared to the results obtained from fitting to all 120 directions of DW-SSFP data acquired at one-flip angle only. The subset of directions was chosen for maximally even coverage in the angular domain, ensuring a fair comparison of an equal number of directions and similar angular resolution between the one- and two-flip angle analyses. The one-vs two-flip angle principal diffusion direction (PDD) estimates were compared using a measure of angular uncertainty from the orientations of samples from the posterior distribution (Jbabdi et al., 2012), defined as a scalar between 0 and 1 (where a larger number corresponds to a higher uncertainty).

### 3.5 Comparisons of tractography pathways acquired with one- and two-flip angle acquisitions

To compare the performance of tractography between the one- and two-flip angle acquisitions, a time-matched comparison was performed using the same data subsets as described in the previous section. To perform tractography, a ball & sticks model (details in Appendix Eq. [A3] and Eq. [A4]) was fit to the DW-SSFP data using cuDIMOT. The two-flip angle fitting was similarly constrained for the ball & sticks model as with the tensor model (described in *Data Processing*) with a shared set of stick orientations and unique diffusivity estimates acquired at each flip angle. Probabilistic tractography was performed using PROBTRACKX2 (Behrens et al., 2007; Hernandez-Fernandez et al., 2019) (step length ​= ​0.5 ​mm, no. samples ​= ​5000, curvature threshold ​= ​0.2) over both the cingulum bundle and the anterior segment of the corpus callosum associated with fiber projections into the prefrontal cortex. These two pathways were chosen because they cover areas of the white matter where either flip angle has lower contrast to noise.

For the cingulum bundle, tracts were seeded in the dorsal segment of the cingulum bundle (CBD) in both the left and right hemispheres. Masks were generated by transforming the pre-defined CBD masks from XTRACT (De Groot et al., 2013; Warrington et al., 2019) into the space of the post-mortems brain using ANTS (Avants et al., 2011). The transformed CBD masks were subsequently defined over white matter only by multiplying by the white matter mask of each brain. To prevent fibers crossing across the two hemispheres, a sagittal exclusion mask was defined over the entire midline of each postmortem brain.

For the anterior segment of the corpus callosum, a standard space mask of the corpus callosum was split into five segments using a previously proposed segmentation scheme (Hofer and Frahm, 2006). The anterior mask associated with fiber projections into the prefrontal cortex (region 1 in (Hofer and Frahm, 2006)) was transformed into the space of the post-mortem brains using ANTS (Avants et al., 2011). The transformed anterior callosum masks were subsequently defined over white matter only by multiplying by the white matter mask of each brain. To ensure that fibers projected into the pre-frontal cortex, an coronal inclusion mask was defined anteriorly to the callosal mask.

### 3.6 Combination of eigenvalue estimates at two-flip angles to a single effective b-value

Eigenvalue estimates at a single effective b-value were estimated using the full set of 120 DW-SSFP directions obtained at both flip angles. Fitting a tensor model to the experimental data (as described in *Methods – Data Processing*), a shared set of ${\vec{V}}_{1,2,3}$ and unique $L_{1,2,3}$ were estimated at each flip angle. Fitting was performed as described in *Theory: Extension to a tensor model* in order to determine voxelwise $D_{m}$ and $D_{s}$ estimates along ${\vec{V}}_{1,2,3}$ (Eq. [4]- setting λ=1). The estimated $D_{m_{1,2,3}}$ and $D_{s_{1,2,3}}$ maps were subsequently substituted into Eq. [3] to generate $L_{1,2,3}$ maps in terms of a single ${\text{b}}_{\text{eff}}$.

Fitting was performed using the eigenvalue estimates (output from the cuDIMOT model) at each flip angle. Voxelwise estimates of T_1_, T_2_ and B_1_ were incorporated into this fitting process as fixed parameters. Fitting was performed in Python using SciPy *curve_fit*, implemented with the Levenberg-Marquardt algorithm (Levenberg, 1944) and accelerated using the Numba compiler (Lam et al., 2015). The integral in Eq. [1] was evaluated using SciPy *quad*.

### 3.7 Choice of ${\text{b}}_{\text{eff}}$

${\text{b}}_{\text{eff}}$ can be chosen to account for the variable SNR of the $L_{1,2,3}$ estimates over the entire brain to produce SNR-optimal results. One approach to achieve this is described in the Supplementary Material (Supplementary Material: *Determination of an SNR-optimal* $b_{eff}$). At the superior and inferior edges of the brain (areas of low B_1_), this SNR-optimal b-value would require an interpolation that relies heavily on the $\alpha_{\text{low}}$ dataset. However, in these regions it was found that the $\alpha_{\text{low}}$ dataset is extremely noisy (due to the low flip angle - Fig. 1b).

The resulting noise amplification was sufficiently problematic that the ADCs could not be characterised in these regions of the $\alpha_{\text{low}}$ datasets, requiring an additional constraint (Supplementary Material Fig. S2). Due to this, we decided that a more pragmatic approach for our acquired data was to select the effective b-value that closely corresponds to low B_1_ regions of the $\alpha_{\text{high}}$ datasets, minimising the interpolation/extrapolation of eigenvalue estimates from this value within these regions.

**Fig. 6:**
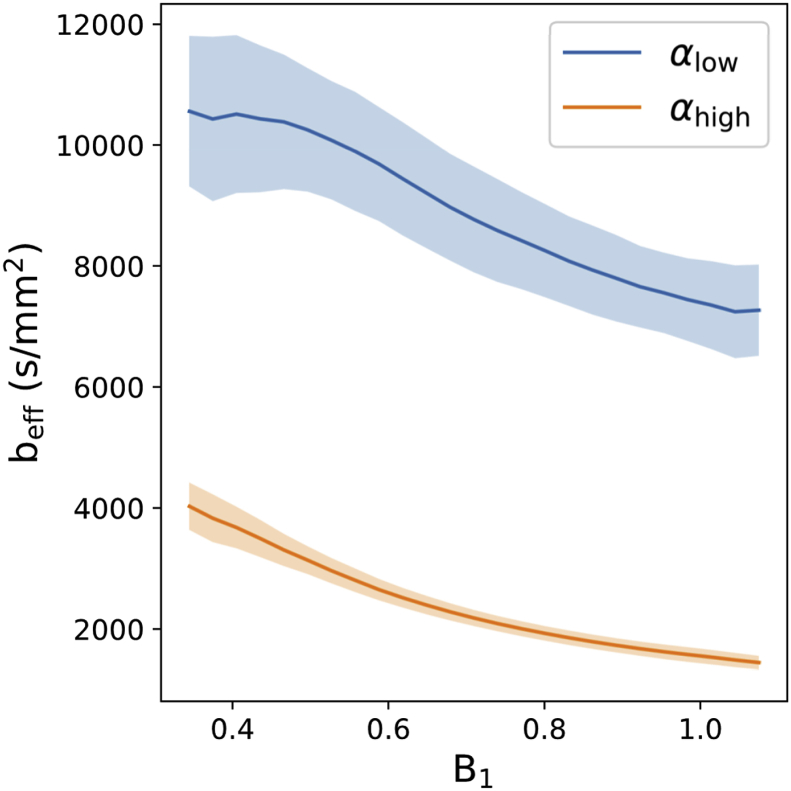
$b_{eff}$**as a function of B**_**1**_**.** Over all 5 brains, the effective b-value increases with decreasing B_1_. The effective b-values estimated at $\alpha_{\text{low}}$ (blue lines) are consistently higher than those estimated at $\alpha_{\text{high}}$ (orange lines), consistent with an expectation of an increased ${\text{b}}_{\text{eff}}$ with decreased flip angle (Fig. 5c). ${\text{b}}_{\text{eff}}=4000\;\text{s}/{\text{mm}}^{2}$ corresponds to the approximate ${\text{b}}_{\text{eff}}$ in areas of low B_1_ at $\alpha_{\text{high}}$ (orange line – left). Here the solid lines display the mean ${\text{b}}_{\text{eff}}$ across all five brains, with the error bars displaying the standard deviation across all five brains.

To estimate ${\text{b}}_{\text{eff}}$ within these regions, voxelwise ${\text{b}}_{\text{eff}}$ maps were generated from the $D_{m_{1}}$, $D_{s_{1}}$ and $L_{1}$ maps, by fitting with Eq. [3]. Example ${\text{b}}_{\text{eff}}$ maps are displayed in Fig. 3c and d. Fig. 6 reveals the effective b-value estimated as a function of B_1_ over all five brains. From examination of Fig. 6, ${\text{b}}_{\text{eff}}=4000\;\text{s}/{\text{mm}}^{2}$ was determined as the effective b-value that closely corresponds to regions of low B_1_ for the $\alpha_{\text{high}}$ dataset.

## 4 Results

### 4.1 Comparison of PDD estimates acquired with one- and two-flip angle acquisitions

**Fig. 7:**
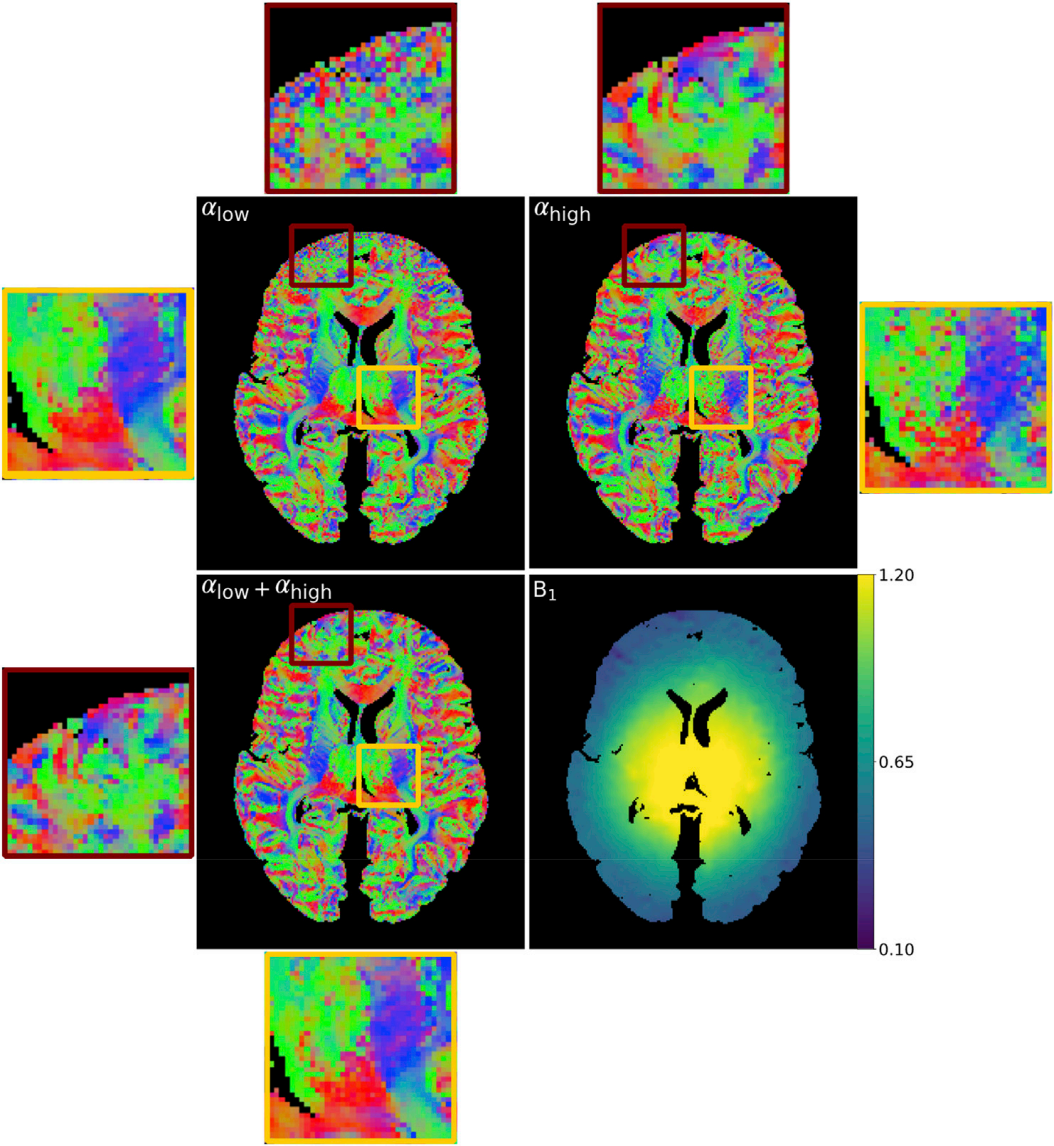
**Visual comparison of the PDD estimates.** At $\alpha_{\text{low}}$, B_1_ inhomogeneity leads to incoherent PDD estimates near the brain boundary (red box), with coherent PDD estimates near the centre of the brain (orange box). At $\alpha_{\text{high}}$, the converse is true. By fitting with two-flip angles ($\alpha_{\text{low}}+\alpha_{\text{high}}$), we obtain a good compromise between the low and high flip angle datasets, yielding coherent PDD estimates over the entire brain.

The benefit of the time-matched two-flip angle approach for overcoming B_1_ dependent CNR in PDD estimates is illustrated in Fig. 7. PDD estimates derived from data acquired at $\alpha_{\text{low}}$ (120 directions) display greater coherence between voxels near the centre of the brain (Fig. 7 orange box – $\alpha_{\text{low}}$). As the scanner sets the nominal flip angle (24°) to be matched to this region, we expect the CNR to be maximized (as predicted in Fig. 1b). Within this region, clear delineation of the striations within the internal capsule are visible. In this same region, the PDD estimates at $\alpha_{\text{high}}$ (120 directions) are less coherent (Fig. 7 orange box – $\alpha_{\text{high}}$). At the brain boundary where the actual flip angle is far below the nominal flip angle, the opposite is true. The PDD estimates at $\alpha_{\text{high}}$ reveal clear depiction of cortical folding patterns (Fig. 7 red box - $\alpha_{\text{high}}$), which are corrupted by noise at $\alpha_{\text{low}}$ (Fig. 7 red box - $\alpha_{\text{low}}$). In comparison, PDD estimates of the two-flip angle data (120 directions, 60 directions at $\alpha_{\text{low}}$ and 60 directions at $\alpha_{\text{high}}$) (Fig. 7 - $\alpha_{\text{low}}+\alpha_{\text{high}}$) demonstrate that regionally dependent benefits associated with each single-flip analysis are captured by the two-flip angle approach. In this combined scan time-matched dataset, it is possible to visualize cortical folding, whilst maintaining the striations within the internal capsule.

**Fig. 8:**
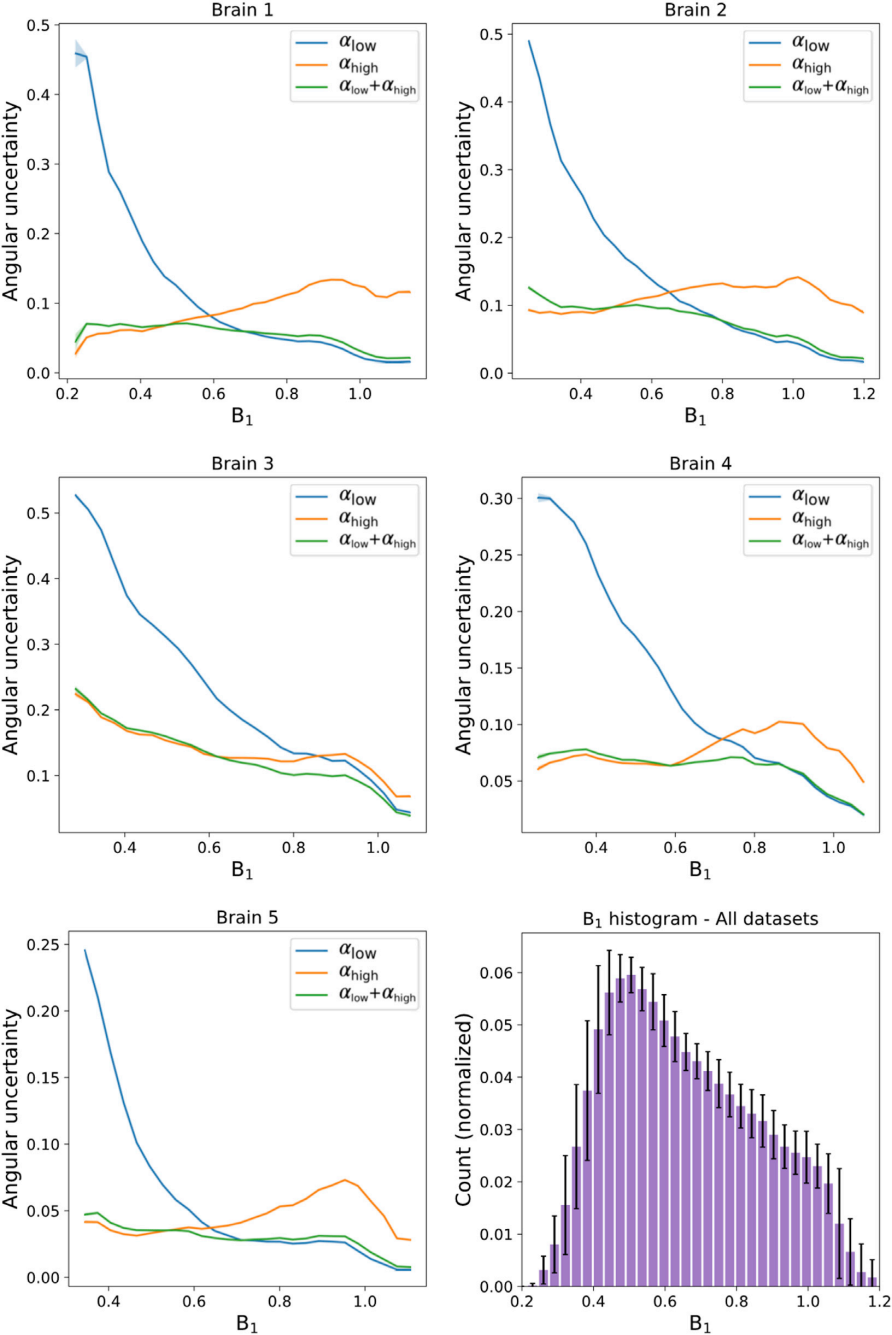
**Quantitative comparison of PDD angular uncertainty vs B**_**1**_**.** In all 5 brains, it can be seen that PDD angular uncertainty estimates are reduced in areas of low/high B_1_ for the $\alpha_{\text{high}}$/$\alpha_{\text{low}}$ datasets respectively. After the proposed combination of two-flip angle data ($\alpha_{\text{low}}+\alpha_{\text{high}}$), the PDD uncertainty estimates are close to those of the single-flip angle within their respective regions of high CNR across the entire range of B_1_. Between these values (where the blue and orange lines cross), the dual-flip approach generates PDD estimates with a reduced angular uncertainty. Plots generated in white matter only from the PDD uncertainty and B_1_ maps for each of the five datasets. The standard error of PDD dispersion values are plotted for each brain, but due to the large number of points per bin these error bars are not visible across most of the plot. The B_1_ histogram (bottom right) reveals that the B_1_ values sampled within these datasets spans a wide range of B_1_, with error bars denoting the standard deviation over the five datasets.

Fig. 8 shows how the angular uncertainty varies as a function of B_1_, where low uncertainty indicates high CNR. In all five datasets, the low B_1_ near the periphery of the brain leads to a higher angular uncertainly in the $\alpha_{\text{low}}$ datasets when compared to those acquired at $\alpha_{\text{high}}$. In areas of high B_1_ the opposite is true, in agreement with Fig. 7. The dual-flip approach ($\alpha_{\text{low}}+\alpha_{\text{high}}$) is able to generate PDD estimates with angular uncertainty close to the best performance obtained for the one-flip angle datasets at the extremes of high or low B_1_, and outperforms either single-flip dataset between these values (i.e. where the curves cross in Fig. 8). A histogram (Fig. 8, bottom right) shows the broad range of B_1_ values sampled in our post-mortem brains.

**Fig. 9:**
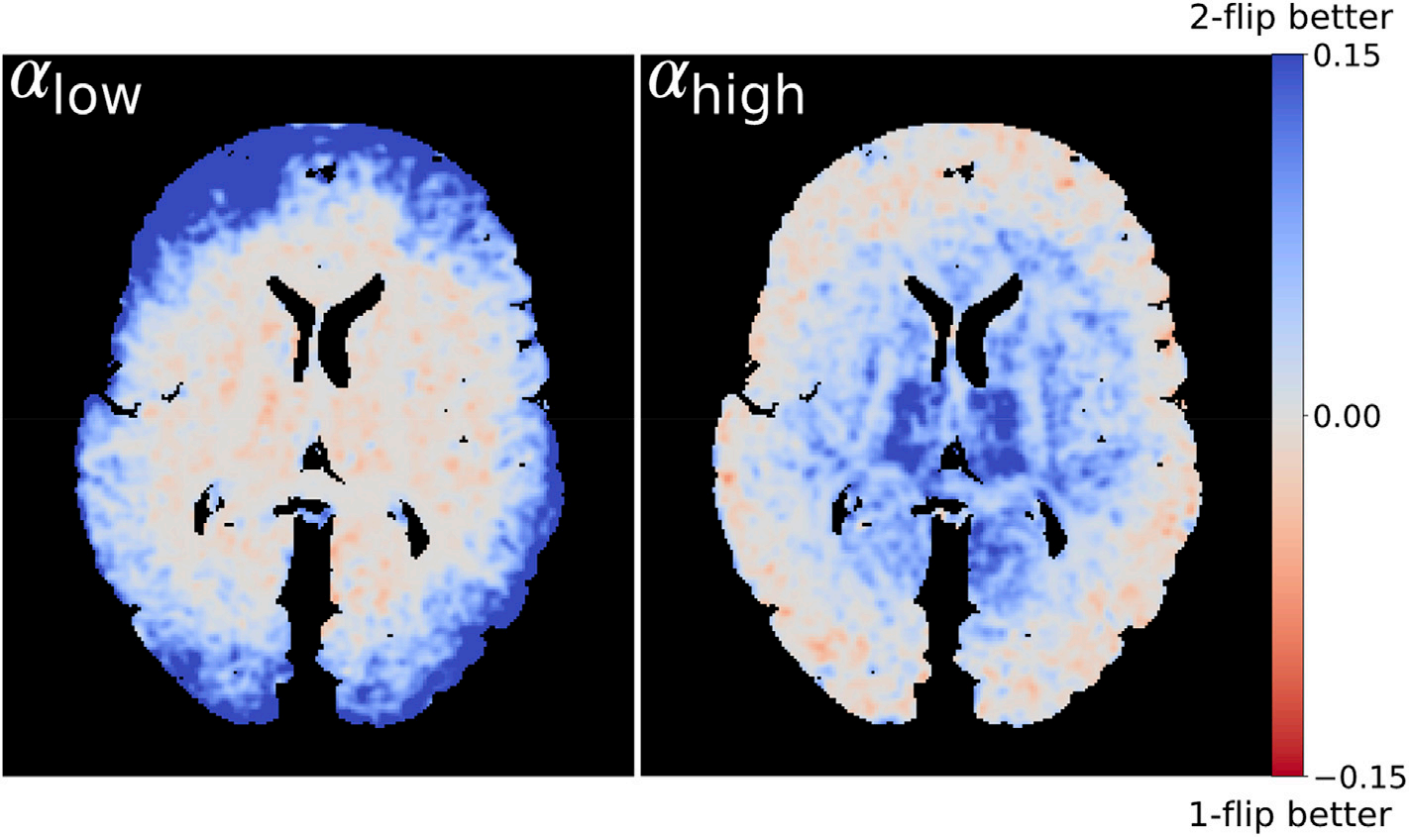
**Visual comparison of the differences in PDD angular uncertainty.** Positive values (blue) display regions where the two-flip angle approach outperforms the single-flip angle, whereas negative values (red) display the opposite. Areas of higher/lower uncertainty are in good visual agreement with the coherence of the PDD estimates in Fig. 7. To aid visualization, the uncertainty differences were smoothed with a Gaussian filter (standard deviation ​= ​1 ​mm).

**Fig. 10:**
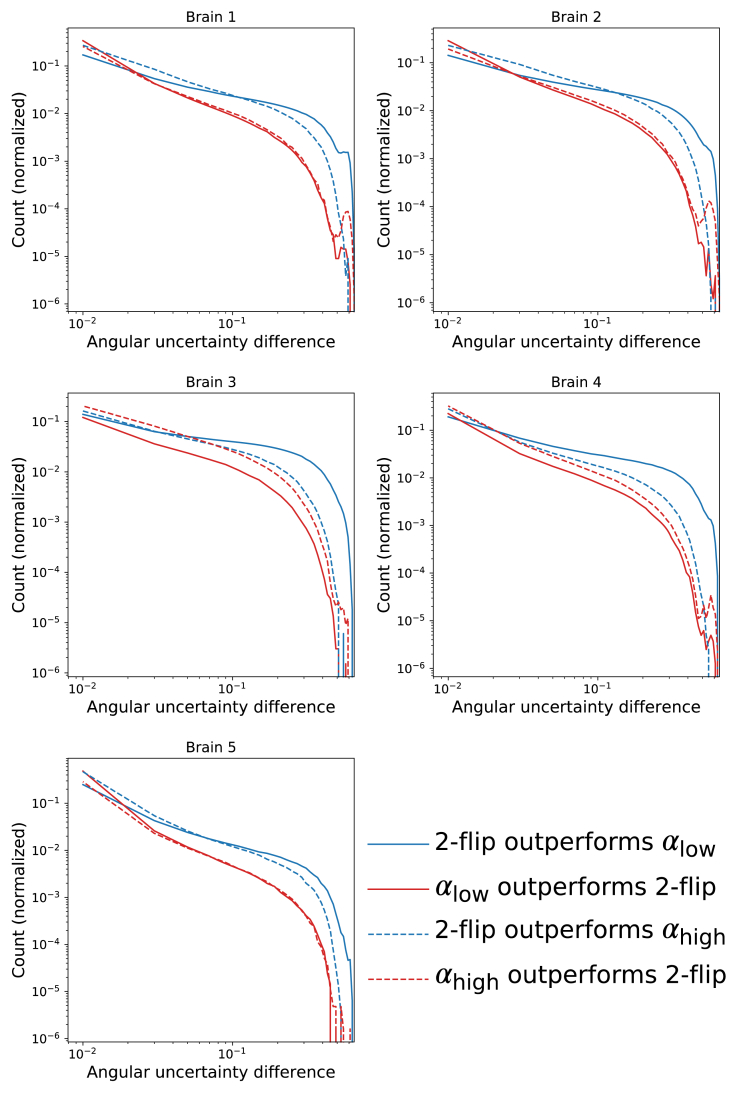
**Quantitative comparison of the differences in PDD angular uncertainty.** These PDD uncertainty difference histograms represent the number of voxels where the one-/two-flip angle PDD estimates outperforms the other. Here, solid/dashed lines refer to the difference between the single ($\alpha_{\text{low}}$/$\alpha_{\text{high}}$) and the dual flip angle approach ($\alpha_{\text{low}}+\alpha_{\text{high}}$) respectively. Blue lines indicate the number of voxels where the two-flip angle approach outperforms the single-flip angle, whereas the red lines display the opposite. A log scale is used on both the x- and y-axes.

Fig. 9 shows a map of the difference in uncertainty between the one- and two-flip angle results. While there are parts of the brain where acquisition at a single, optimal flip angle provides slightly lower uncertainty compared to the two-flip angle approach (light red), over the entire dataset the dual-flip approach provides a net gain (dark blue). By creating a histogram of the difference in PDD angular uncertainty between the one- and two-flip angle analyses (Fig. 10), we can see an increased fraction of voxels with the two-flip angle approach that have a reduction in uncertainty in comparison to the single flip approaches (blue curves above red). The opposite is true for small differences in angular uncertainty (red curves above blue) and a few voxels with very high angular uncertainty. The overall improvements in angular uncertainty for the two-flip angle approach vs $\alpha_{\text{high}}$ are reduced in comparison to $\alpha_{\text{low}}$, reflecting the large number of voxels at $\alpha_{\text{low}}$ which have high angular uncertainty (Fig. 8).

### 4.2 Comparisons of tractography pathways acquired with one- and two-flip angle acquisitions

**Fig. 11:**
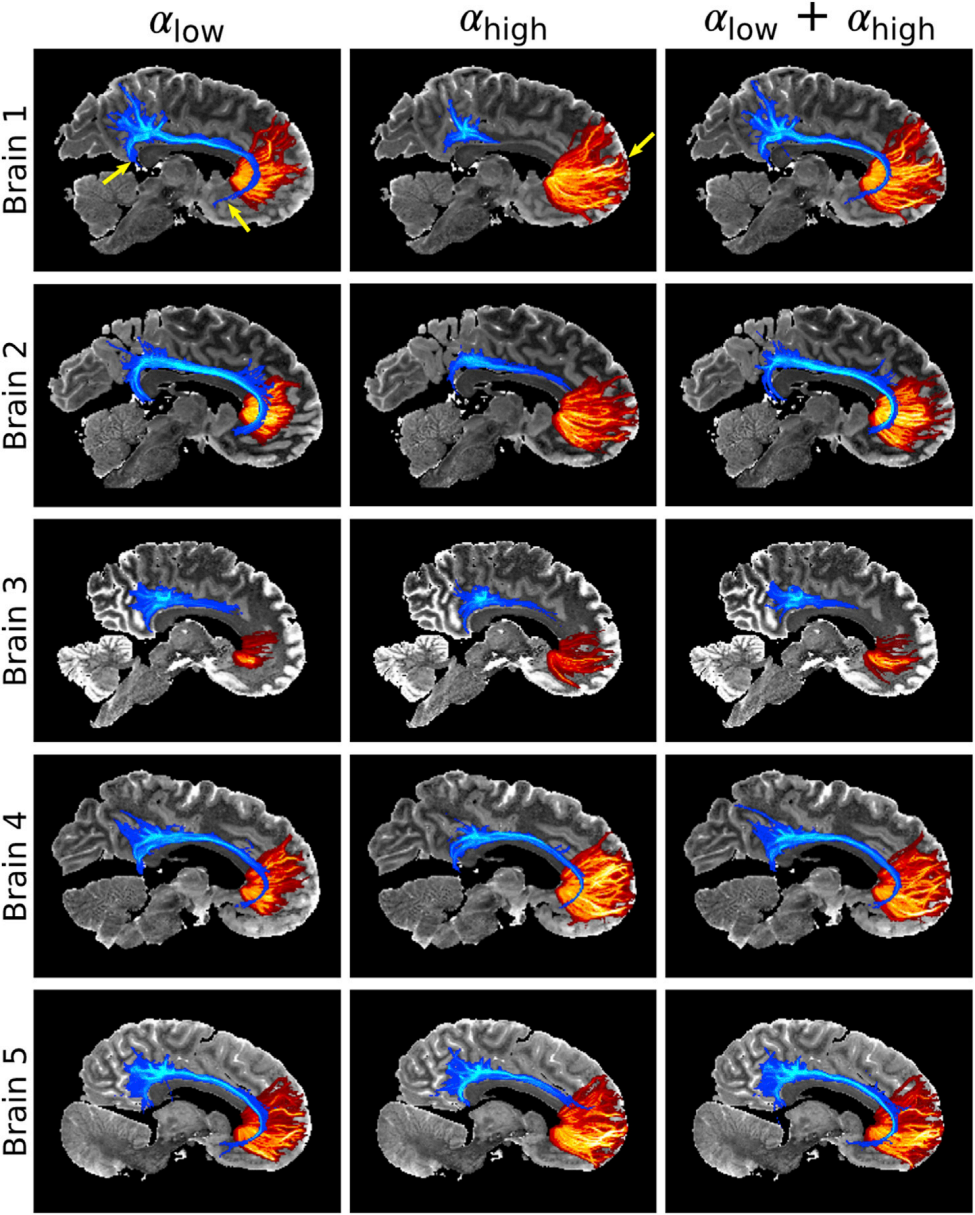
**Tractography comparison between the single and dual flip approaches.** The cingulum bundle is associated with regions of high B_1_. The pre-frontal cortex is associated with regions of low B_1_. The high CNR near the centre of the brain at $\alpha_{\text{low}}$ (left) leads to clearly defined tracts over the cingulum bundle (blue), including posterior projections toward the temporal lobe and anterior projections toward the basal forebrain (Brain 1 - $\alpha_{\text{low}}$ yellow arrows). However, the low SNR in cortical regions leads to poor tractography performance for fibers projecting to the pre-frontal cortex (red). For the $\alpha_{\text{high}}$ datasets (middle), the opposite is true, with a reduced representation of the cingulum bundle but capturing the pre-frontal callosal projections all the way into cortex (Brain 1 - $\alpha_{\text{high}}$ yellow arrow). The dual flip approach (right) leads tractography estimates more consistent with the single flip performance in regions of high CNR. Tractography displayed as maximum-intensity projections (MIP) of the streamline density, with the cingulum bundle MIP overlaid above the pre-frontal cortex MIP in all images. A consistent display range is used for the streamline density across all images, with the cingulum bundle and pre-frontal cortex displayed between $5\cdot 10^{3}$ - $4\cdot 10^{4}$ and $2\cdot 10^{4}$ - $3\cdot 10^{5}$ streamlines respectively.

Tractography streamline density maps estimated from the one and two flip angle acquisitions are displayed in Fig. 11. As the cingulum bundle is close to the centre of the brain, it is associated with areas of high B_1_ (high/low CNR in the $\alpha_{\text{low}}$/$\alpha_{\text{high}}$ datasets respectively). The cingulum bundle tract (blue) spans a greater extent of the brain for the $\alpha_{\text{low}}$ datasets (left) vs $\alpha_{\text{high}}$ (middle). Reconstruction across the whole posterior-anterior extent is achieved for 4/5 brains at $\alpha_{\text{low}}$, and only 1/5 brains at $\alpha_{\text{high}}$. These differences are most apparent in Brain 1, where the data at $\alpha_{\text{high}}$ is unable to reconstruct the cingulum bundle. For the callosal projections to the pre-frontal cortex (red), the opposite is true. Over this pathway, fiber tracts at $\alpha_{\text{low}}$ are visibly shorter than those at $\alpha_{\text{high}}$, with differences most apparent in Brain 2.

The single flip angle acquisitions lead to poor performance in either the cingulum bundle or callosal projections, as these tracts traverse areas of high or low B_1_ respectively. However for the two-flip angle estimates (right), the combination of datasets with high SNR at low and high B_1_ leads to reconstruction of these fiber tracts closer to the best of the two flip angles. A notable exception is Brain 3, where there is poor reconstruction of the two pathways in all three datasets. Brain 3 has a higher angular uncertainty than the other four brains (Fig. 8), associated with an overall lower data quality.

### 4.3 Combination of eigenvalue estimates at two-flip angles to a single ${\text{b}}_{\text{eff}}$

**Fig. 12:**
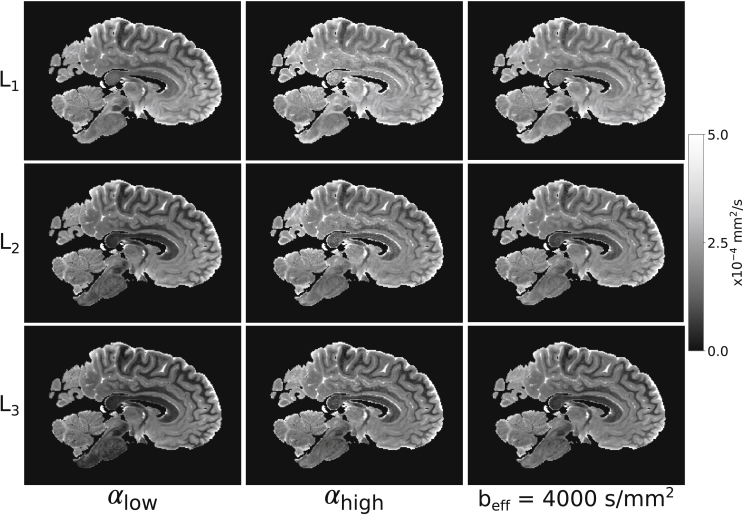
**Visual comparison of the**$L_{\mathrm{1,2,3}}$**estimates.** Differences in the $L_{1,2,3}$ maps at each flip angle agrees with the expectation that within a non-Gaussian regime, an increased flip angle in DW-SSFP yields higher diffusivity estimates (Fig. 5c). The $L_{1,2,3}$ maps at ${\text{b}}_{\text{eff}}=4000\text{ s}/{\text{mm}}^{2}$ reveal improved SNR vs $\alpha_{\text{high}}$ and more homogenous diffusivity estimates over tissue.

**Fig. 13:**
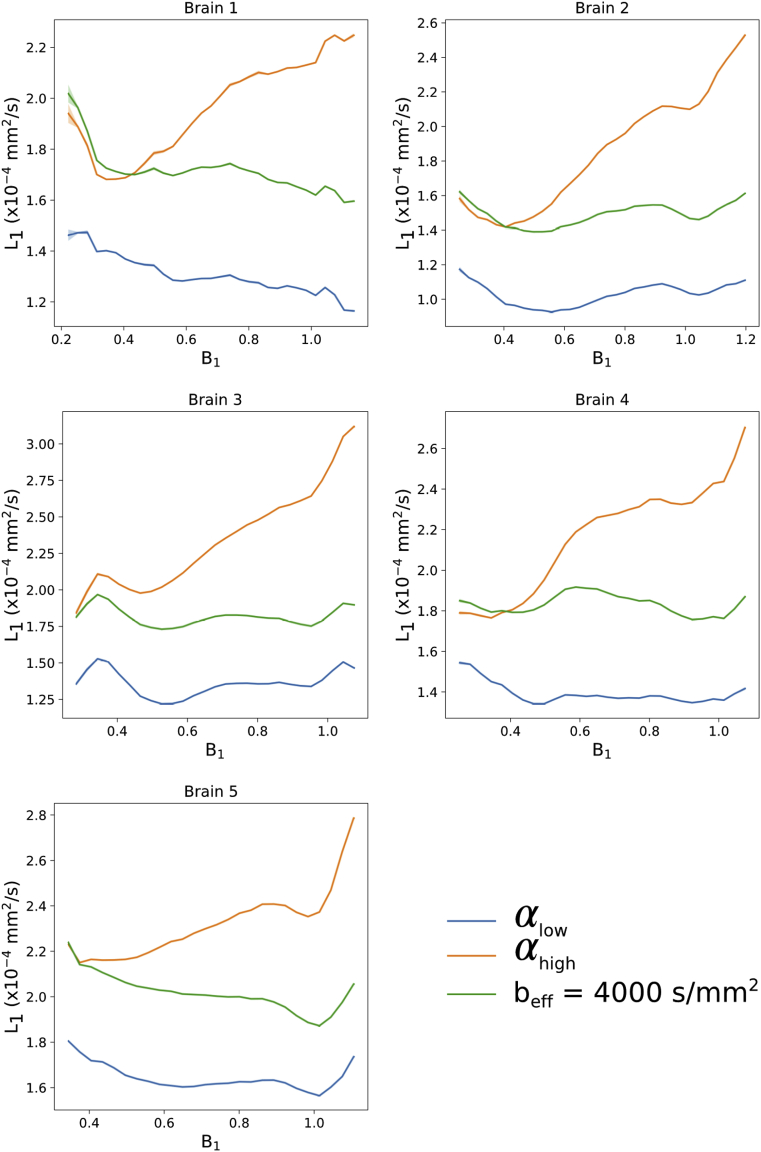
**Quantitative comparison of**$L_{1}$**estimates vs B**_**1**_**.** Here we observe an increased $L_{1}$ estimate in DW-SSFP data acquired at $\alpha_{\text{high}}$, in agreement with (Tendler et al., 2020) and Fig. 5c. The $L_{1}$ estimates at ${\text{b}}_{\text{eff}}=4000\text{ ​s}/{\text{mm}}^{2}$ display a flatter distribution, consistent with removal of the influence of B_1_. Plots generated in white matter only from the $L_{1}$ and B_1_ maps for each of the five datasets. The standard error of $L_{1}$ estimates within each bin are plotted for each brain, but due to the large number of points per bin these error bars are not visible across most of the plot.

$L_{1,2,3}$ estimates calculated from DW-SSFP data at each flip angle (Fig. 12) display observable differences in the derived diffusivity values, overall showing an increased diffusivity estimate at $\alpha_{\text{high}}$ (confirmed in Fig. 13). Previous work (Tendler et al., 2020) makes clear that effective b-values are overall higher with lower flip angles, which would be consistent with these variations in diffusivity being driven by restriction in tissue. Furthermore, this indicates that we cannot simply average the eigenvalue estimates acquired at different DW-SSFP flip angles, as it would combine maps with distinct ADC estimates at each flip angle.$\;L_{1,2,3}$ maps at ${\text{b}}_{\text{eff}}=4000\text{ ​s}/{\text{mm}}^{2}$ show reduced inhomogeneity, and improved SNR compared to $\alpha_{\text{high}}$.

As shown in Fig. 13, the reconstructed $L_{1}$ estimates at ${\text{b}}_{\text{eff}}=4000\text{ ​s}/{\text{mm}}^{2}$ give good agreement to the $\alpha_{\text{high}}$ results at low B_1_, whilst maintaining a flat distribution across all five brains. The crossing point of the $L_{1}$ curves at $\alpha_{\text{high}}$ and ${\text{b}}_{\text{eff}}=4000\text{ ​s}/{\text{mm}}^{2}$ reveals the approximate flip angle along $L_{1}$ where ${\text{b}}_{\text{eff}}=4000\text{ ​s}/{\text{mm}}^{2}$.

**Fig. 14:**
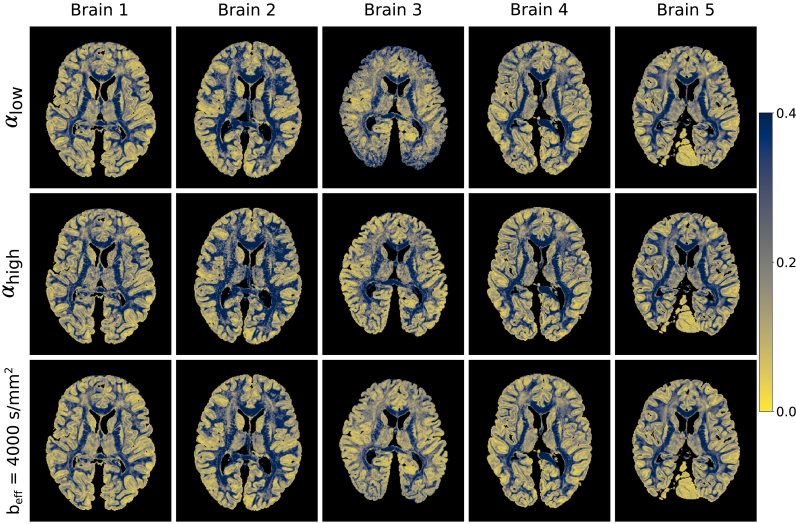
**Visual comparison of FA.** Differences between the FA maps at each flip angle are consistent with differences in non-Gaussianity along the three tensor eigenvectors, additionally revealing a reduced SNR in the derived FA maps near the boundary/centre at $\alpha_{\text{low}}$/$\alpha_{\text{high}}$ respectively (most apparent in Brains 3 and 4). The FA maps at ${\text{b}}_{\text{eff}}=4000\text{ s}/{\text{mm}}^{2}$ yield more consistent SNR across the tissue. Colormap chosen to highlight the variable contrast and noise over the brain.

**Fig. 15:**
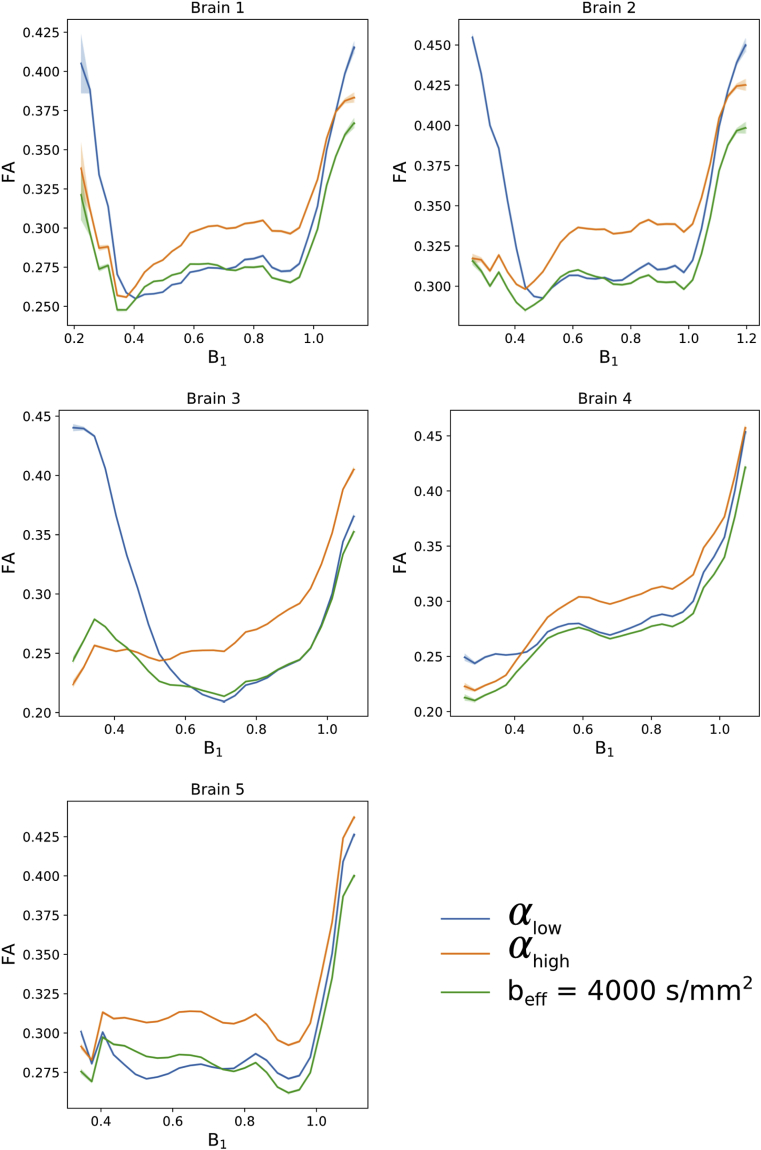
**Quantitative comparison of FA estimates vs B**_**1.**_ Here we observe a difference in the FA estimates from DW-SSFP data at each flip angle, consistent with variations in the non-Gaussian properties of tissue along the estimated eigenvalues. Plots generated in white matter only from the FA and B_1_ maps for each of the five datasets. The standard error of FA estimates within each bin are plotted for each brain, but due to the large number of points per bin these error bars are not visible across most of the plot.

Fractional anisotropy (FA) maps over all five brains (Fig. 14) additionally display differences in the estimated FA at each flip angle (confirmed in Fig. 15), consistent with restriction along $L_{1,2,3}$. These FA maps have an increased sensitivity to noise in comparison to the $L_{1,2,3}$ estimates and the FA maps derived from DW-SSFP data at $\alpha_{\text{low}}$/$\alpha_{\text{high}}$ have lower SNR at the edge/centre of the brain respectively, consistent with the PDD results in Fig. 7. The FA maps generated at ${\text{b}}_{\text{eff}}=4000\text{ ​s}/{\text{mm}}^{2}\text{ ​}$do not reveal the same spatial variation, yielding high SNR across the brain. The impact of B_1_ is displayed in Fig. 15.

## 5 Discussion

This work demonstrates how the effects of B_1_ inhomogeneity in DW-SSFP can be accounted for by using data acquired at two-flip angles and an appropriate signal model that captures non-Gaussian diffusion. By utilizing a pair of prescribed flip angles that optimize CNR across a range of B_1_, we provide a means to obtain a homogeneous and interpretable characterization of diffusion across the brain. We demonstrate the potential of this approach by quantifying the spatial profile of angular uncertainty in PDD estimates and diffusivity estimates as a function of B_1_.

Previous work (Foxley et al., 2014a) demonstrated that with a one-flip angle DW-SSFP acquisition, angular uncertainty in PDD estimates was reduced by increasing field strength from 3T to 7T, providing motivation to move to higher field when performing tractography. This reduction in uncertainty would be expected in local regions of tissue due to the higher SNR associated with an increase in field strength, but would be mitigated by the B_1_ effects considered in this work (Fig. 7). Using the two-flip approach described in this paper, PDD estimates at 7T can be obtained over whole post-mortem brain samples (Fig. 7), reducing the number of voxels with high angular uncertainty in tissue regions that experience a sub-optimal flip angle (Fig. 10).

Given the pattern of B_1_ and the need for high quality data in central white matter for tractography, the dual flip approach should provide a particular benefit for tractography into the grey matter. The dual flip approach does not restrict tract reconstructions to specific areas of the brain (Fig. 11) associated with high or low B_1_. Such data provides a means to reconstruct fiber projections towards the cortex, in addition to tracts that span the centre of brain.

For these post-mortem brain samples, SNR-optimal estimates are predicted to be achieved at a low flip angles. An SNR-optimal ${\text{b}}_{\text{eff}}$ corresponds to an approximate flip angle of 21° - 28° (Supplementary Material Fig. S3d), achieved at B_1_ values of 0.88 - 1.17/0.22 - 0.30 for the $\alpha_{\text{low}}$/$\alpha_{\text{high}}$ datasets. The plots in Fig. 8 show that the two-flip angle approach achieves an angular uncertainty estimate close to the single flip angle approach in these B_1_ regions and performs better between these B_1_ values.

An increased estimate of ADC at higher flip-angles (Fig. 12, Fig. 13) demonstrates deviations of the DW-SSFP signal from the Buxton model, consistent with a model of restriction and the results in (Tendler et al., 2020). Our correction reduces the variation of ADC with B_1_ (Fig. 13), in addition to modifying the distribution of derived metrics such as FA (Fig. 15). This allows for more accurate comparisons of diffusivity estimates within different brain regions. Furthermore, as the B_1_ distribution is not reliably calibrated at scan time, our approach allows for comparison of diffusivity estimates between different post-mortem brain samples. The divergence of the $\alpha_{\text{high}}$ and ${\text{b}}_{\text{eff}}=4000\text{ ​s}/{\text{mm}}^{2}$ plots (Fig. 13), emphasizes the influence of B_1_ on measured ADC.

The FA maps in Fig. 14 reveal the trend of reduced SNR at $\alpha_{\text{low}}$/$\alpha_{\text{high}}$ near the centre/edge of the brain, consistent with the PDD (Fig. 7) maps. However, the very low SNR/CNR in areas of low B_1_ in the $\alpha_{\text{low}}$ dataset lead to spurious diffusivity estimates, requiring the incorporation of a diffusivity constraint (Supplementary Material: *Constraint for the dual-flip approach due to regions of low signal*). Due to this, we chose to interpolate to an effective b-value which corresponded to areas of low B_1_ in the $\alpha_{\text{high}}$ dataset, where diffusivity estimates can be reliably estimated. Our original optimization (Fig. 2) did not include regions of very low B_1_, which is likely to have contributed to a choice of flip angles that provided very low SNR/CNR within these regions. As the SNR/CNR drops off very sharply at low flip angles (as can be inferred from Fig. 1b), a nominal flip angle for the low flip angle dataset greater than 24° would have reduced the volume of tissue where diffusivity estimates could not be reliably estimated.

An alternative approach to correct for the influence of B_1_ would be to use a parallel transmit system at acquisition, to improve the homogeneity of B_1_ across the brain. This requires the use of specialist equipment and can be challenging to implement, but is compatible with the DW-SSFP method. A homogenous B_1_ field does not mitigate some of the other challenges associated with DW-SSFP, most notably that the effective b-value also depends on the T_1_ and T_2_ of the imaged tissue (as can be inferred from the ${\text{b}}_{\text{eff}}$ maps in Fig. 3c and d). Moreover, acquisition of DW-SSFP data at a single flip angle, even with parallel transmit, would not provide a means to model the effects of non-Gaussianity within tissue to generate maps at a single effective b-value. Our approach provides a means to resolve both of these challenges, and could be further improved with a parallel transmit system to increase the SNR of the resultant images. However, our approach does require the acquisition of two DW-SSFP datasets per direction, necessitating longer scans to obtain a given number of directions (or achieving fewer directions in a fixed scan duration). Since datasets acquired at different flip angles are characterised by different signal amplitudes/diffusion contrast, acquiring two datasets per direction does not simply translate into a $\sqrt{2}$ increase in SNR (reducing the SNR efficiency), though may provide a means to investigate non-Gaussianity within tissue (Tendler et al., 2020).

Despite the challenges associated with DW-SSFP, our use of the DW-SSFP sequence is motivated by a very high SNR efficiency. Previous work has demonstrated the benefits of DW-SSFP vs DW-SE when imaging fixed post-mortem tissue at 3T (Miller et al., 2012), where the short T_2_ and low diffusivity of fixed, post-mortem tissue leads to challenges when imaging with DW-SE. This is due to the requirement of both fast acquisition (due to the short T_2_) and a high b-value (due to the low diffusivity), which cannot be easily achieved on whole-body MR systems with conventional gradient sets. The further reduction in T_2_ at ultra-high field exacerbates these problems with DW-SE, whereas the DW-SSFP sequence has demonstrated improved SNR at 7T vs 3T (Foxley et al., 2014a).

DW-STEAM mitigates the effects of T_2_ relaxation and can achieve high b-values by increasing the diffusion time during T_1_ relaxation (Fritz et al., 2019). In comparison to DW-SSFP, DW-STEAM is characterised by a simpler diffusion model and additionally does not require the acquisition of dependency datasets (T_1_, T_2_ and B_1_ maps) to accurately estimate diffusion coefficients. However, the signal forming mechanisms of DW-STEAM lead to a twofold reduction in the available signal that must be balanced against these gains. Moreover, increasing b-value via longer diffusion times in DW-STEAM is less efficient than increasing the b-value through the diffusion gradient (quadratic increase with gradient time compared to linear increase with diffusion time). The signal forming mechanisms of DW-SSFP predict an improved SNR efficiency vs DW-STEAM. However, the requirement of 2x data acquisition with the approach outlined in this manuscript will mitigate some of these gains in SNR-efficiency. The relative pros and cons of these sequence must be carefully considered and to date a direct comparison in these post-mortem samples have not been performed.

In this work, we used a two-step approach to estimate the gamma distribution parameters ($D_{m_{1,2,3}}$ and $D_{s_{1,2,3}}$) from DW-SSFP data acquired at two flip angles, first estimating the eigenvalues at each flip angle and then fitting the gamma parameters to these eigenvalue estimates. An alternative would be to use a kurtosis model, however this would require many more free parameters to explicitly model the kurtosis covariance. Our two-step approach provides a simple method to estimate each gamma distribution independently, where along the eigenvectors there is no covariance between the gamma distributions.

In this work, we utilised the Buxton model of DW-SSFP (Buxton, 1993) to investigate the diffusivity properties of tissue. An alternative DW-SSFP signal model is the Freed model (Freed et al., 2001), which has been shown to provide more accurate estimates of the DW-SSFP signal under certain experimental regimes. Recent work (Tendler et al., 2020) additionally demonstrated that the Freed model provides greater agreement to Monte-Carlo simulations of the DW-SSFP signal compared to the Buxton model assuming a gamma distribution to model non-Gaussianity. In the parameter regime and sample properties explored within this study, the Buxton and Freed models (assuming a gamma distribution of diffusivities) predict very similar signal contrast and attenuation (Supplementary Material Fig. S4) across a wide range of B_1_, indicating that we would expect to estimate similar diffusivity estimates for the two models investigated and is sufficient for our analysis. However, our approach is not restricted to the Buxton model and could be readily incorporated into alternative models such as the Freed model.

This study was motivated by the interest in understanding whether diffusivity could provide biomarkers that are related to neuropathology in ALS. This necessitates measures of diffusivity in post-mortem tissue that can be compared to histopathological stains. To be meaningful, these diffusivity measures need to be driven primarily by the underlying tissue (as reflected in restrictions that cause non-Gaussian behaviour) rather than confounds like B_1_ inhomogeneity. For example, neurodegenerative diseases such as ALS have been shown to reduce FA *in vivo* (Agosta et al., 2010). A more consistent measurement of FA across white matter, obtained from results at a single ${\text{b}}_{\text{eff}}$ (Fig. 14) would allow for more accurate measurements in post-mortem data to corroborate *in vivo* findings. Future work that directly compares diffusivity to histology will consider whether there is evidence for a neuropathological signature in diffusion MRI.

## 6 Conclusion

DW-SSFP at 7T has the potential to provide high signal and contrast diffusion weighted imaging in post-mortem tissue. However, B_1_ inhomogeneity coupled with the dependence of diffusion contrast on flip angle means that the resulting signal is not straightforward to interpret. We proposed to use a multi-flip angle DW-SSFP acquisition alongside a non-Gaussian signal model to account for B_1_ inhomogeneity at 7T. With this method, we can obtain improved estimates of diffusion properties within tissue, including both quantitative diffusivities and fiber orientations.

## CRediT authorship contribution statement

**Benjamin C. Tendler:** Conceptualization, Methodology, Software, Formal analysis, Writing - original draft, Writing - review & editing. **Sean Foxley:** Conceptualization, Methodology, Software, Investigation, Writing - original draft. **Moises Hernandez-Fernandez:** Software. **Michiel Cottaar:** Methodology. **Connor Scott:** Resources. **Olaf Ansorge:** Resources. **Karla L. Miller:** Conceptualization, Methodology, Investigation, Writing - review & editing, Supervision. **Saad Jbabdi:** Conceptualization, Methodology, Software, Writing - review & editing, Supervision.

## Declaration of competing interest

None.

## Appendix

### Buxton model definitions

The Buxton model of DW-SSFP is defined as:

[A1] $$S_{\text{SSFP}}\left(\alpha ,T_{1},T_{2},\text{TR},q,D\right)=-\frac{S_{0}\left(1-E_{1}\right)E_{2}A_{2}^{-\frac{2}{3}}\left(F_{1}-E_{2}A_{1}A_{2}^{\frac{2}{3}}\right)\sin\;\alpha }{r-F_{1}s},$$

with:

$$E_{1}=e^{-\frac{\text{TR}}{T_{1}}},$$

$$E_{2}=e^{-\frac{\text{TR}}{T_{2}}},$$

$$A_{1}=e^{-q^{2}\cdot \text{TR}\cdot D},\;\;A_{2}=e^{-q^{2}\cdot \tau \cdot D},$$

q=γGτ,

$$r=1-E_{1}\;\cos\;\alpha +E_{2}^{2}A_{1}A_{2}^{1/3}\left(\cos\;\alpha -E_{1}\right),$$

$$s=E_{2}A_{1}A_{2}^{-4/3}\left(1-E_{1}\;\cos\;\alpha \right)+E_{2}A_{2}^{-\frac{1}{3}}\left(\cos\;\alpha -E_{1}\right),$$

$$F_{1}=K-{\left(K^{2}-A_{2}^{2}\right)}^{1/2},$$

$$K=\frac{1-E_{1}A_{1}\;\cos\;\alpha -E_{2}^{2}A_{1}^{2}A_{2}^{-2/3}\left(E_{1}A_{1}-\cos\;\alpha \right)}{E_{2}A_{1}A_{2}^{-4/3}\left(1+\cos\;\alpha \right)\left(1-E_{1}A_{1}\right)}.$$

where:

α - Flip angle,
$T_{1}$ and $T_{2}$ - Relaxation times,
TR - Repetition time,
γ - Gyromagnetic ratio,
G - Diffusion gradient amplitude,
τ - Diffusion gradient duration,
$S_{0}$ - Equilibrium magnetization,
D – Diffusion coefficient,

### Buxton model diffusion tensor

A diffusion tensor is incorporated into the DW-SSFP Buxton signal model (Eq. [A1]) by defining:

[A2] $$A_{1}=e^{-q^{2}\cdot \text{TR}\cdot {\vec{g}}^{T}\mathrm{VL}V^{T}\vec{g}},\;A_{2}=e^{-q^{2}\cdot \tau \cdot {\vec{g}}^{T}\mathrm{VL}V^{T}\vec{g}},$$

where $\vec{g}$ is the orientation of the diffusion gradient, V is the eigenvector matrix $\left[\vec{V_{1}},\vec{V_{2}}\;,\vec{V_{3}}\right]$ and L is the eigenvalue matrix $\left[\begin{array}{lll} L_{1} & 0 & 0\\ 0 & L_{2} & 0\\ 0 & 0 & L_{3} \end{array}\right]$.

### Buxton model ball & sticks

A ball & sticks model is incorporated into the DW-SSFP Buxton signal model (Eq. [A1]) by defining:

[A3] $$S_{{\text{SSFP}}_{b\&s}}=\left(1-\sum_{j=1}^{2}f_{j}\right)\cdot S_{{\text{SSFP}}_{\text{iso}}}+\sum_{j=1}^{2}S_{\text{j},{\text{SSFP}}_{\text{aniso}}},$$

where $f_{j}$ defines the signal contribution from stick j, $S_{{\text{SSFP}}_{\text{iso}}}$ is equivalent to Eq. [A1] and for $S_{\text{j},{\text{SSFP}}_{\text{aniso}}}$ we define (from Eq. [A1]):

[A4] $$A_{1}=e^{-q^{2}\cdot \text{TR}\cdot D\cdot {\left({\vec{g}}^{T}{\vec{\mu }}_{j}\right)}^{2}},\;A_{2}=e^{-q^{2}\cdot \tau \cdot D\cdot {\left({\vec{g}}^{T}{\vec{\mu }}_{j}\right)}^{2}},$$

where $\vec{g}$ is the orientation of the diffusion gradient and ${\vec{\mu }}_{j}$is the j-th stick orientation (Behrens et al., 2007).
