# Supplementary material for: Use of multi-flip angle measurements to account for transmit inhomogeneity and non-Gaussian diffusion in DW-SSFP
Source: Neuroimage. 2020 Oct 15;220:117113. doi: 10.1016/j.neuroimage.2020.117113 (PMC7573656; doi:10.1016/j.neuroimage.2020.117113)
Supplement: Multimedia component 1 [file mmc1.pdf]

Supplementary Material

White matter masks

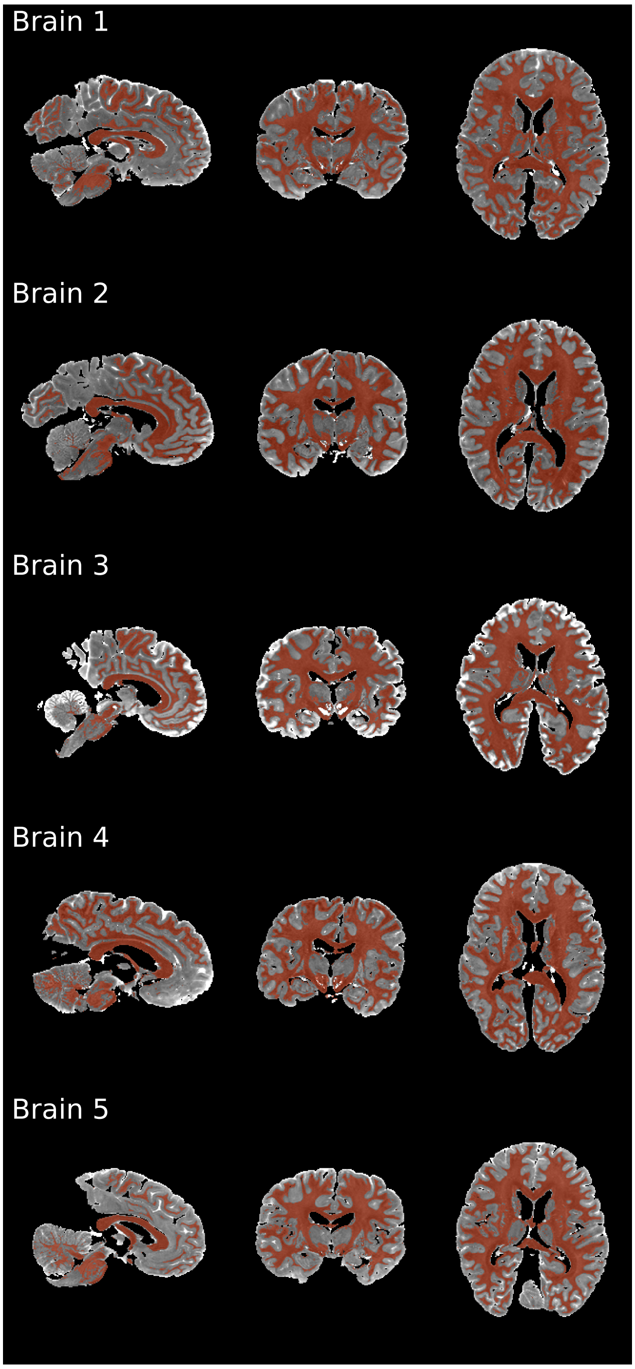

Supplementary Figure S1: White matter masks (red) for all five brains. All 5 maps were generated using FSL FAST (Zhang et al., 2001) from the mean diffusivity maps estimated at $b_{eff} = 4000\ s/mm^2$ (which display strong grey-white matter contrast). To generate these masks, six tissue classes were generated per brain. The binary classes that corresponded to white matter were combined. Remaining holes within the white matter mask were filled using *fslmaths -fillh* option (available in the FSL toolbox). For Brain 4, the partial volume estimation segmentation maps corresponding to white matter were additionally incorporated into the white matter mask.

Constraint for the dual-flip approach due to regions of low signal

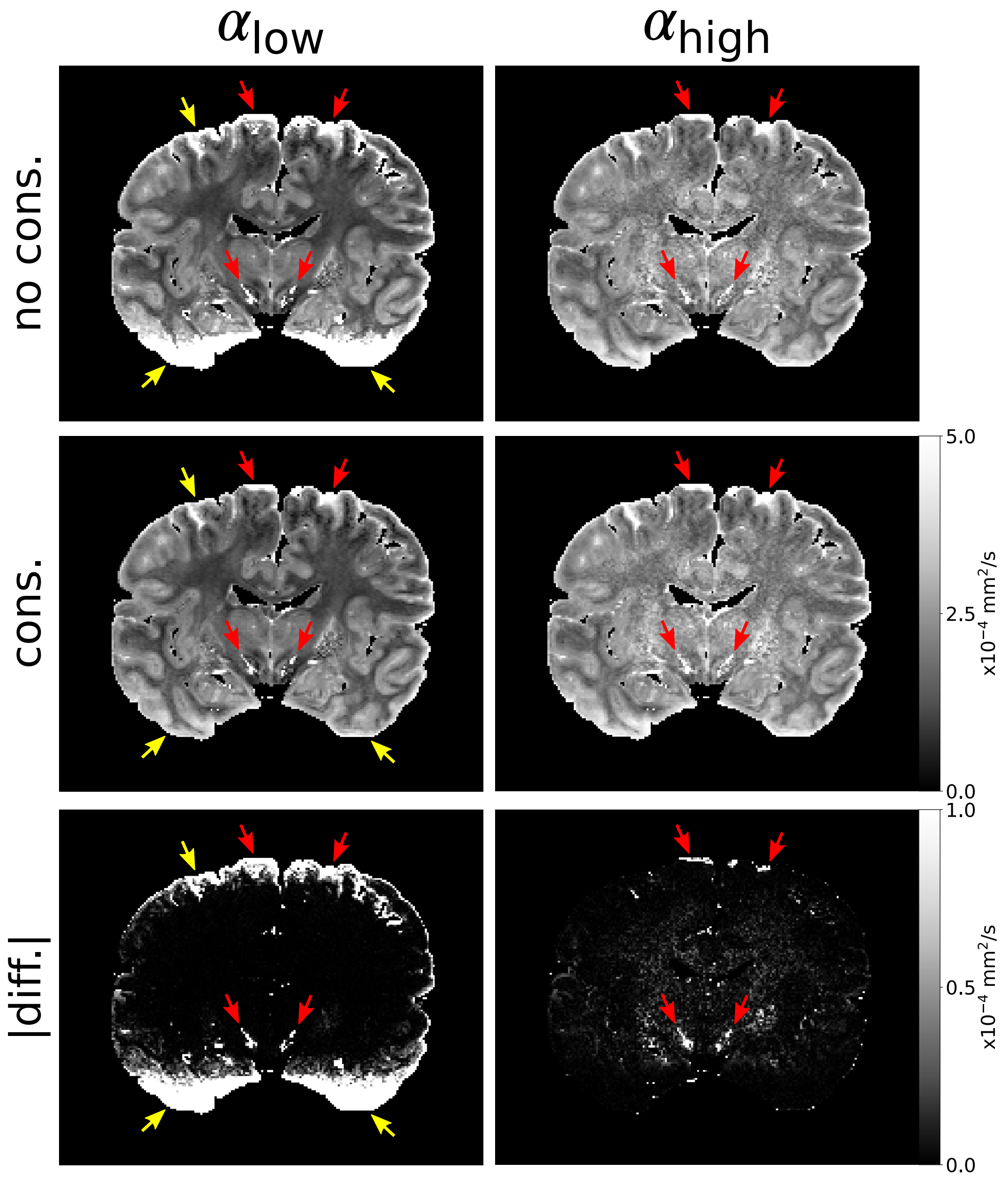

Supplementary Figure S2: – L_1 diffusivity estimates for a single postmortem brain at each flip angle, comparing the dual-flip angle fitting with (middle) and without (top) the diffusivity constraint (ensuring that the voxelwise diffusivity estimates obtained at α_{low} must be lower than the diffusivity estimates at α_{high}). Areas of low B_1 in the α_{low} dataset are associated with very low contrast and signal levels (Figs. 1b and 2 – Main Text). Within these regions, the fitting process was unable to rectify the diffusivity estimates, leading to spuriously high values (yellow arrows). The diffusivity constraint sets an upper bound to diffusivity estimates within these regions (middle). The absolute difference maps (bottom – scaled between 0 and $1 \cdot 10^{-4}$ mm^2/s) demonstrate that the differences are predominantly associated to these regions, with small changes in other areas also associated with low diffusion contrast. High diffusivity estimates remain in areas where there is low signal in both the α_{low} and α_{high} datasets (red arrows).

An earlier version of this work did not incorporate the noise-floor fitting (Eq. [5] – Main Text). However, after implementation of the noise-floor, it was observed that within regions of the α_{low} dataset associated with low B_1, the signal levels were close to (or at) the noise-floor. Within these regions the tensor model was unable to provide an accurate fit. This lead to spuriously high diffusivity estimates (Fig. S2 – top row). To account for this in the dual-flip approach, we leveraged the information available from the other flip angle. Given two diffusivity estimates per voxel, we added the constraint that the voxelwise diffusivity estimates obtained at α_{low} must be lower than the diffusivity estimates at α_{high}. This is analogous to constraining diffusivity estimates from a DW-SE experiment at a lower b-value to be greater than a diffusivity estimate obtained at a higher b-value. The correction (Fig S2 - middle row) brings the diffusivity estimates within these regions in line with other regions of tissue, with small changes (Fig. S2 – bottom row) within other regions associated with low diffusion contrast.

Determination of an SNR-optimal b_{eff}

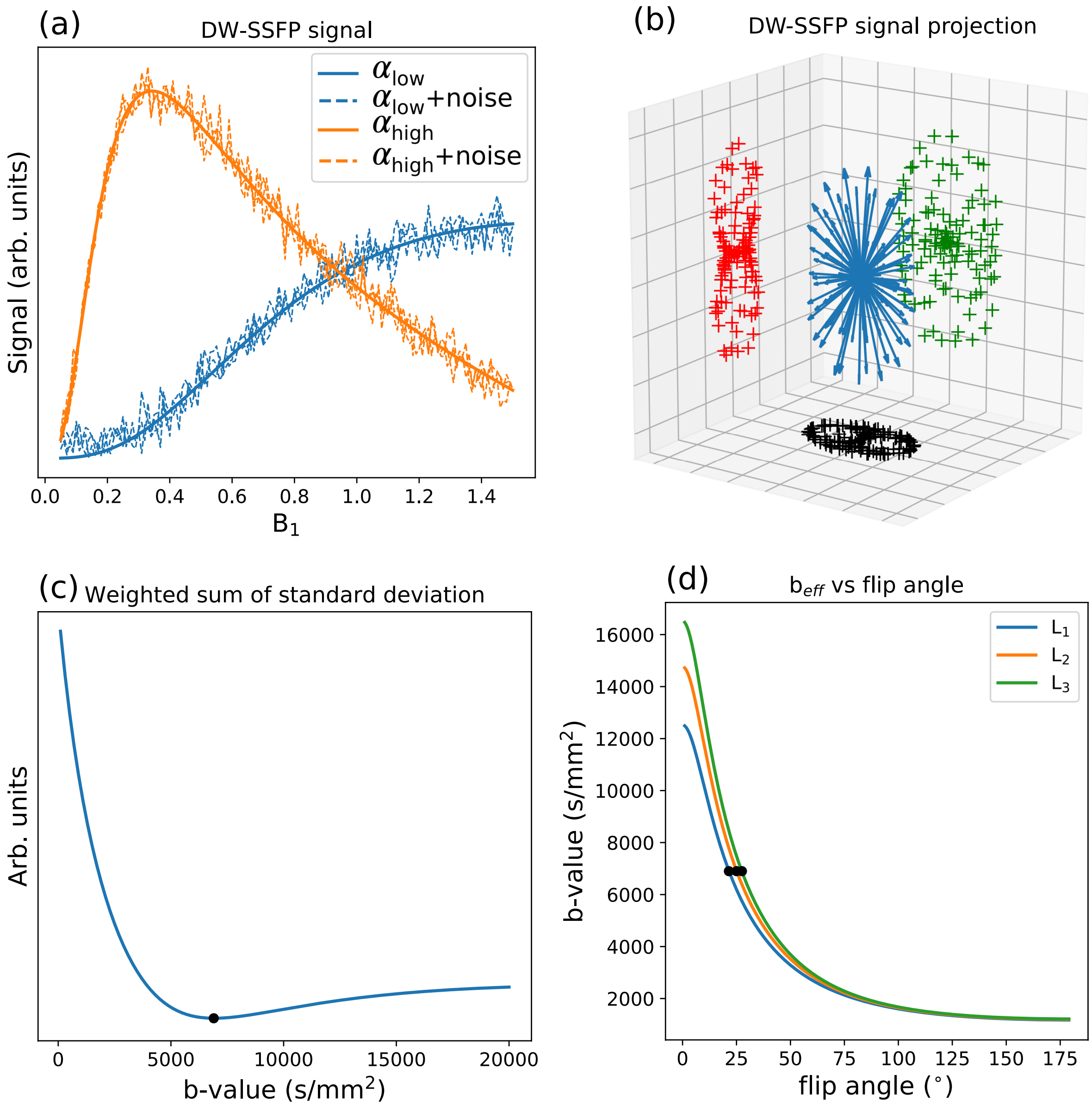

Supplementary Figure S3: (a) Example DW-SSFP signal curve simulated at nominal flip angles 24° and 94° across a range of B_1. The dashed lines display example signal curves with added Rician noise. (b) Example distribution of the DW-SSFP signal simulated over the 120 diffusion directions. To highlight the anisotropy of the distribution, the baseline signal common to all 120 diffusion directions was subtracted. (c) Results of the optimization to determine an SNR-optimal b_{eff}, determined as $b_{eff} = 6900\ s/mm^2$ (black dot). (d) Simulating the b_{eff} over a range of flip angles, our SNR-optimal b_{eff} corresponds to a flip angle of ~ 21° − 28° over $L_{1,2,3}$.

Theory

Given a set of sample properties and DW-SSFP sequence parameters, different flip angles lead to different $\mathrm{b_{eff}}$ estimates. Choosing a $\mathrm{b_{eff}}$ that corresponds to a flip angle that yields high SNR DW-SSFP data will lead to SNR-optimal diffusivity estimates.

To determine an SNR-optimal $\mathrm{b_{eff}}$, we investigated how different $\mathrm{b_{eff}}$ estimates affected the SNR of resulting diffusivity maps. This was achieved by simulating the DW-SSFP signal with added Rician noise. To account for the different flip angles sampled across the brain (and the respective SNR/CNR of DW-SSFP data at different flip angles), the distribution of B_1 encountered in our experimental data was incorporated into the analysis. Simulations were designed to closely follow the sample properties and acquisition protocol used in our study.

Method

To determine an SNR optimal $\mathrm{b_{eff}}$, we performed simulations with synthetic DW-SSFP datasets generated at nominal flip angles 24° and 94° over a range of B_1 values with added Rician noise (Fig. S3a). The synthetic datasets were simulated using the Buxton model of DW-SSFP (Buxton, 1993) assuming a diffusion tensor model and a non-Gaussian diffusion estimator defined by a gamma distribution of diffusivities. The synthetic datasets were designed to closely match the properties of the postmortem brain samples and experimental acquisition as follows:

- At each flip angle, DW-SSFP datasets over 120 diffusion directions ($\mathrm{q} = 300\ \mathrm{cm}^{-1}$) and six non-diffusion weighted datasets were simulated. Diffusion directions were generated using the GPS tool in FSL (Jenkinson et al., 2012; Jones et al., 1999).
- To match our experimental protocol, $\mathrm{TR} = 28\ \mathrm{ms}$.
- $\mathrm{T_1} = 567\ \mathrm{ms}$ and $\mathrm{T_2} = 28.7\ \mathrm{ms}$, the mean values over white matter of all five post-mortem brains.
- The datasets were simulated under a non-Gaussian diffusion estimator (gamma distribution of diffusivities), setting $D_{m1} = 2.9 \cdot 10^{-4}\ \mathrm{mm^2/s}$, $D_{m2} = 2.0 \cdot 10^{-4}\ \mathrm{mm^2/s}$, $D_{m3} = 1.5 \cdot 10^{-4}\ \mathrm{mm^2/s}$, $D_{s1} = 3.3 \cdot 10^{-4}\ \mathrm{mm^2/s}$, $D_{s2} = 2.4 \cdot 10^{-4}\ \mathrm{mm^2/s}$ and $D_{s3} = 1.9 \cdot 10^{-4}\ \mathrm{mm^2/s}$ the mean values of D_m and D_s along $L_{1,2,3}$ over white matter estimated from the five post-mortem brains. The

orientations of the eigenvectors $\vec{V}_1$, $\vec{V}_2$ and $\vec{V}_3$ were kept constant for all simulated signals.

- The datasets were simulated over the range $B_1 = 0.05$ to $B_1 = 1.50$, in steps of 0.01. For each B_1 value, 1000 noise repeats were simulated, with the noise level estimated from the repeats of the experimental non-diffusion weighted DW-SSFP data (Fig. S3a).

An example simulated DW-SSFP signal distribution over all 120 directions is displayed in Fig. S3b.

The two-flip angle simulated data were subsequently processed in a similar manner to the two-flip angle experimental data. Unique $L_{1,2,3}$ estimates at each flip angle were generated for each B_1 value and noise repeat (Eqs. [A1] and [A2] – Main Text). $D_{m1,2,3}$ and $D_{s1,2,3}$ were subsequently determined from the $L_{1,2,3}$ estimates (Eq. [1] – Main Text). From the values of $D_{m1,2,3}$ and $D_{s1,2,3}$, $L_{1,2,3}$ estimates were generated for a single $\mathrm{b_{eff}}$, over the range $\mathrm{b_{eff}} = 100\ \mathrm{s/mm^2}$ to $\mathrm{b_{eff}} = 20000\ \mathrm{s/mm^2}$ in steps of $100\ \mathrm{s/mm^2}$ (Eq. [3] – Main Text).

From the $L_{1,2,3}$ estimates simulated at each $\mathrm{b_{eff}}$, the standard deviation was determined over the 1000 noise repeats at each B_1 value. The standard deviations estimated at each B_1 value were subsequently weighted by the B_1 histogram of the experimental data (Fig. 8 – Main Text) and summed. The $\mathrm{b_{eff}}$ with the minimum summed standard deviation was defined as the SNR-optimal $\mathrm{b_{eff}}$.

Results

Figure S3c reveals the distribution of the weighted sum of the standard deviation with $\mathrm{b_{eff}}$, determining the SNR-optimal $\mathrm{b_{eff}} = 6900\ \mathrm{s/mm^2}$ (Fig. S3c – black dot). As our simulations have been performed for a set of fixed parameters, each flip angle will correspond to a unique $\mathrm{b_{eff}}$ along $L_{1,2,3}$. Plotting the relationship between $\mathrm{b_{eff}}$ and flip angle (Fig. S3d), we can determine that the SNR-optimal $\mathrm{b_{eff}}$ corresponds to a flip angle of 21° – 28° (Fig. S3d – black dots).

Freed vs Buxton Model

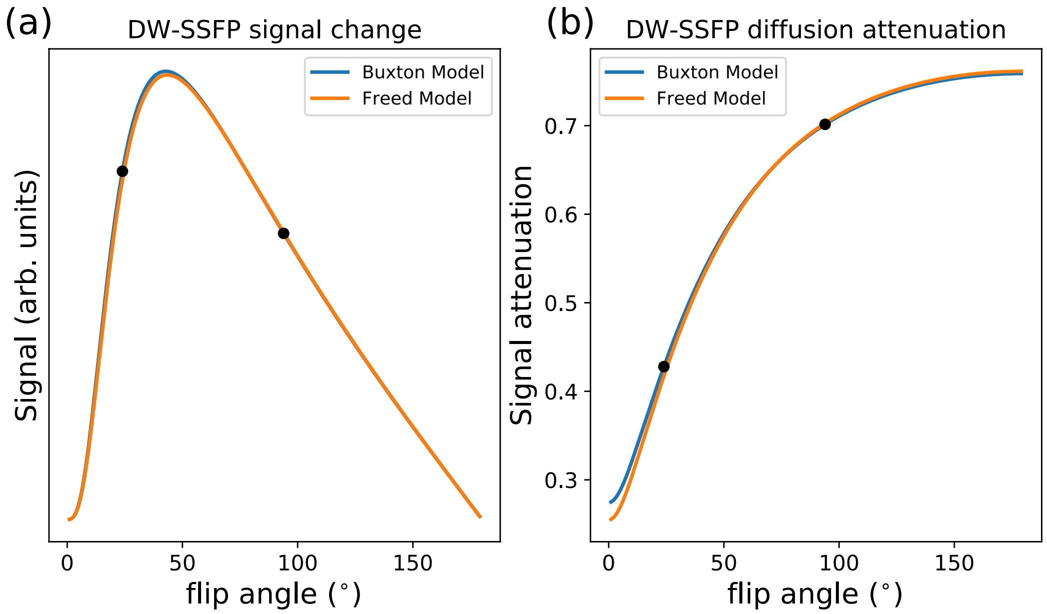

Supplementary Figure S4: Under the experimental regime of the DW-SSFP acquisitions utilized in this study, the DW-SSFP contrast (a) and signal attenuation (b) predicted by the Buxton (Buxton, 1993) and Freed (Freed et al., 2001) models under a gamma distribution of diffusivities reveal very similar signal evolution. The maximum deviation of signal attenuation is estimated at low flip angles, associated with the low B_1 regions of the α_{low} datasets, which typically have low SNR. The black dots indicate the location of the 24° and 94° nominal flip angles used in our study. Simulation performed using the DW-SSFP experimental parameters from our acquisition (Table 1 – Main Text), defining T_1, T_2, D_m and D_s from the T_1, T_2, D_{m1} and D_{s1} maps (estimated as the mean over white matter of all five brains).

References

Buxton, R.B., 1993. The diffusion sensitivity of fast steady-state free precession imaging. Magn. Reson. Med. https://doi.org/10.1002/mrm.1910290212

Freed, D.E., Scheven, U.M., Zielinski, L.J., Sen, P.N., Hürlimann, M.D., 2001. Steady-state free precession experiments and exact treatment of diffusion in a uniform gradient. J. Chem. Phys. https://doi.org/10.1063/1.1389859

Jenkinson, M., Beckmann, C.F., Behrens, T.E.J., Woolrich, M.W., Smith, S.M., 2012. FSL - Review. Neuroimage. https://doi.org/10.1016/j.neuroimage.2011.09.015

Jones, D.K., Horsfield, M.A., Simmons, A., 1999. Optimal strategies for measuring diffusion in anisotropic systems by magnetic resonance imaging. Magn. Reson. Med. https://doi.org/10.1002/(SICI)1522-2594(199909)42:3<515::AID-MRM14>3.0.CO;2-Q

Zhang, Y., Brady, M., Smith, S., 2001. Segmentation of brain MR images through a hidden Markov random field model and the expectation-maximization algorithm. IEEE Trans. Med. Imaging. https://doi.org/10.1109/42.906424
